# Role of e-cigarettes and pharmacotherapy during attempts to quit cigarette smoking: The PATH Study 2013-16

**DOI:** 10.1371/journal.pone.0237938

**Published:** 2020-09-02

**Authors:** John P. Pierce, Tarik Benmarhnia, Ruifeng Chen, Martha White, David B. Abrams, Bridget K. Ambrose, Carlos Blanco, Nicolette Borek, Kelvin Choi, Blair Coleman, Wilson M. Compton, K. Michael Cummings, Cristine D. Delnevo, Tara Elton-Marshall, Maciej L. Goniewicz, Shannon Gravely, Geoffrey T. Fong, Dorothy Hatsukami, James Henrie, Karin A. Kasza, Sheila Kealey, Heather L. Kimmel, Jean Limpert, Raymond S. Niaura, Carolina Ramôa, Eva Sharma, Marushka L. Silveira, Cassandra A. Stanton, Michael B. Steinberg, Ethel Taylor, Maansi Bansal-Travers, Dennis R. Trinidad, Lisa D. Gardner, Andrew Hyland, Samir Soneji, Karen Messer

**Affiliations:** 1 Moores Cancer Center, University of California San Diego, San Diego, California, United States of America; 2 Department of Family Medicine and Public Health, University of California San Diego, San Diego, California, United States of America; 3 Department of Social and Behavioral Sciences, NYU College of Global Public Health, New York University, New York, New York, United States of America; 4 Office of Science, Center for Tobacco Products, U.S. Food and Drug Administration, Silver Spring, Maryland, United States of America; 5 National Institute on Drug Abuse, National Institutes of Health, Bethesda, Maryland, United States of America; 6 National Institute for Minority Health and Health Disparities, National Institutes of Health, Bethesda, Maryland, United States of America; 7 Department of Psychiatry and Behavioral Sciences, Medical University of South Carolina, Charleston, South Carolina, United States of America; 8 Rutgers Center for Tobacco Studies, New Brunswick, New Jersey, United States of America; 9 Institute for Mental Health Policy Research, Centre for Addiction and Mental Health, London, Ontario, Canada; 10 Department of Health Behavior, Roswell Park Comprehensive Cancer Center, Buffalo, New York, United States of America; 11 Department of Psychology, University of Waterloo, Waterloo, Ontario, Canada; 12 Department of Psychology and School of Public Health and Health Systems, University of Waterloo, Waterloo, Ontario, Canada; 13 Ontario Institute for Cancer Research, MaRS Centre, Toronto, Ontario, Canada; 14 Department of Psychiatry, University of Minnesota, Minneapolis, Minnesota, United States of America; 15 Westat, Rockville, Maryland, United States of America; 16 Kelly Government Solutions, Rockville, Maryland, United States of America; 17 Department of Health Behavior, Gillings School of Global Public Health, Chapel Hill, North Carolina, United States of America; University of Wisconsin-Madison, UNITED STATES

## Abstract

**Background:**

More smokers report using e-cigarettes to help them quit than FDA-approved pharmacotherapy.

**Objective:**

To assess the association of e-cigarettes with future abstinence from cigarette and tobacco use.

**Design:**

Cohort study of US sample, with annual follow-up.

**Participants:**

US adult (ages 18+) daily cigarette smokers identified at Wave 1 (W1; 2013–14) of the PATH Study, who reported a quit attempt before W2 and completed W3 (n = 2443).

**Exposures:**

Use of e-cigarettes, pharmacotherapy (including nicotine replacement therapy), or no product for last quit attempt (LQA), and current daily e-cigarette use at W2.

**Analysis:**

Propensity score matching (PSM) of groups using different methods to quit.

**Outcome measures:**

12+ months abstinence at W3 from cigarettes and from all tobacco (including e-cigarettes). 30+ days abstinence at W3 was a secondary outcome.

**Results:**

Among daily smokers with an LQA, 23.5% used e-cigarettes, 19.3% used pharmacotherapy only (including NRT) and 57.2% used no product. Cigarette abstinence for 12+ months at W3 was ~10% in each group. Half of the cigarette abstainers in the e-cigarette group were using e-cigarettes at W3. Different methods to help quitting had statistically comparable 12+ month cigarette abstinence at W3 (e-cigarettes vs no product: Risk Difference (RD) = 0.01, 95% CI: -0.04 to 0.06; e-cigarettes vs pharmacotherapy: RD = 0.02, 95% CI:-0.04 to 0.09). Likewise, daily e-cigarette users at W2 did not show a cessation benefit over comparable no-e-cigarette users and this finding was robust to sensitivity analyses. Abstinence for 30+ days at W3 was also similar across products.

**Limitations:**

The frequency of e-cigarette use during the LQA was not assessed, nor was it possible to assess continuous abstinence from the LQA.

**Conclusion:**

Among US daily smokers who quit cigarettes in 2014–15, use of e-cigarettes in that attempt compared to approved cessation aids or no products showed similar abstinence rates 1–2 years later.

## Introduction

Preventing and reducing use of cigarettes and all tobacco is a major public health goal [1, 2]. While no e-cigarettes have been approved by the Food and Drug Administration (FDA) for smoking cessation to date, adult smokers may try e-cigarettes more frequently than FDA-approved pharmacotherapy aids [3]. A number of systematic reviews [4–7] as well as reports in 2018 from the U.S. National Academies’ of Sciences, Engineering, and Medicine (NASEM) [8], Public Health England [9], and the 2020 Surgeon-General’s report [10] have focused on the potential role of e-cigarettes in cigarette smoking cessation. A lack of consistent findings highlights the need for more evidence. An important point is the suggestive evidence [8, 10] that daily e-cigarette use is more likely than non-daily use to be associated with increased smoking cessation. While a recent randomized trial [11] in the United Kingdom indicated that e-cigarettes were more efficacious in achieving 12+ months cigarette abstinence than nicotine replacement therapy (NRT), longitudinal population-level studies are needed to identify whether the use of e-cigarettes as a method to quit cigarettes is associated with increased successful cigarette cessation at the population level.

The Population Assessment of Tobacco and Health (PATH) Study [12] is a large, nationally representative longitudinal study that collects detailed measures of tobacco use and related information annually. Using the first two waves of the PATH Study, Benmarhnia et. al. [13] reported that using e-cigarettes to help quit smoking was associated with higher concurrent cigarette abstinence, although non-daily cigarette smokers were included so the results may not generalize to daily smokers. Two other studies leveraging PATH data [14, 15] reported that daily e-cigarette use was associated with an increase in smoking cessation. However, neither restricted their sample to those who made a quit attempt, as recommended by NASEM [8], and hence introduced significant bias into the assessment [16]. As the PATH Study did not measure frequency of e-cigarette use for the last quit attempt (LQA), Berry’s analysis [14] assumed that those who made a recent quit attempt using e-cigarettes and who still used them daily at W2, were representative of all daily e-cigarette users at the time of the quit attempt. However, this group excludes many quitters who relapsed between the LQA and W2, and thus stopped using e-cigarettes daily, potentially leading to an upward bias [17] in the observed cessation at W2.

Observational studies do not randomly assign individuals to an exposure, and characteristics associated with self-selection may confound analyses [8, 18] We used propensity score matching (PSM) [19] to adjust for non-comparability between those who used different methods to quit. PSM is a statistical technique used to reduce bias due to confounding variables in observational studies. PSM seeks to obtain comparable exposed and unexposed subjects by matching each subject in the exposed group with unexposed controls, using a set of *a priori* identified potential confounders. For each e-cigarette user, PSM aims to find a closely matched smoker who did not use e-cigarettes to quit and compare average cessation outcomes to account for confounding variables. Thus, PSM allows differences in the outcome of interest to be more likely attributed to the exposure of interest.

**Fig 1:**
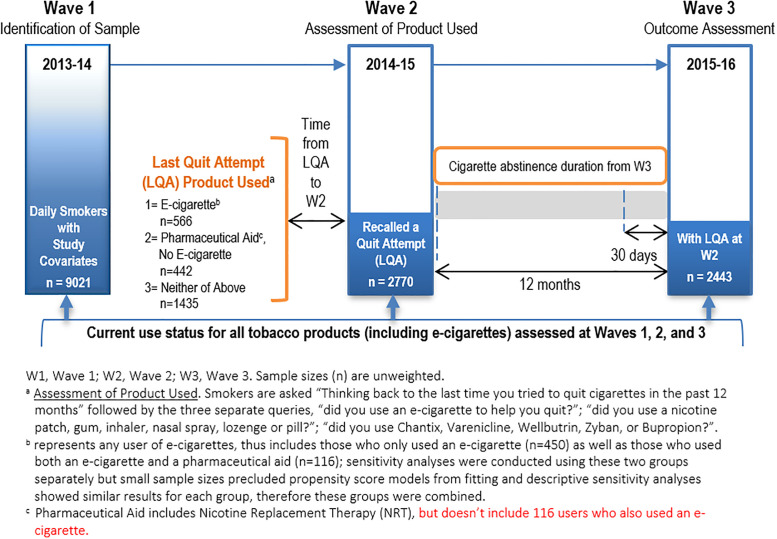
Data collection schema to investigate the role of e-cigarettes and pharmacotherapy during attempts to quit cigarette smoking in the PATH Study.

We used data from PATH Study Wave 1 (W1) adult (18+ years) daily cigarette smokers who, at W2, reported a quit attempt in the past year, as well as the method used to quit on the LQA (Fig 1). We address the following hypotheses:

**Hypothesis 1**: Use of ENDS to help quit during the LQA will be associated with greater abstinence duration at Wave 3 for a) cigarettes and b) all tobacco products, compared to PSM controls who did not use ENDS to help quit during the target LQA.
**Hypothesis 2**: Those who were daily users of ENDS at Wave 2 (.i.e. at some time after the start of their LQA) will be more likely to be cigarette abstinent at Wave 3 than non-e-cigarette users who were similarly successful at the same point in the quit attempt.

As secondary analyses for both hypotheses, we will also use PSM controls who used no products to help quit on the target LQA, and PSM controls who used pharmacotherapy but not ENDS to help quit on the target LQA as comparison.

## Methods

### Data source

The PATH Study is conducted by the National Institute on Drug Abuse, partnering with the Food and Drug Administration’s Center for Tobacco Products, through a contract with Westat. Audio computer-assisted self-interviews (ACASI) in English and Spanish assess tobacco use and associated health behaviors, with data collection in their place of residence at approximately annual intervals. W1 (2013–14) used a stratified, address-based, area-probability sample, oversampling for adult tobacco users, young adults (ages 18 to 24 years), and African-American adults. In the first wave, the PATH Study interviewed 32,320 adults with approximately equal distribution of men and women. There were 19,299 non-Hispanic Whites, 4,496 non-Hispanic Blacks, 5,536 Hispanics, 966 Asians and 1,269 from non-Hispanic multiracial groups. Response rates, based on participation at W1, were: household screener, 54.0%; adult interview W1, 74%; 1-year follow-up (W2, 2014–15), 83.2%; and 2-year follow-up (W3, 2015–16), 78.4%. Study methodology is described at https://doi.org/10.3886/Series606. Further details regarding the PATH Study design and methods have been previously published [12]. The Westat Institutional Review Board approved the study, and all respondents aged 18+ years provided written informed consent.

### Tobacco use

At W1, W2, and W3, the PATH Study assessed ever use and current use (every day, somedays or not at all) of the following tobacco products: cigarettes, e-cigarettes, cigars, pipes, hookah and smokeless products. For each product, ever users who responded “not at all” to the current use question were asked, “About how long has it been since you last smoked/used [product]?” At W2, all product users were asked, ““In the past 12 months, have you stopped [product] use for one day or longer because you were trying to quit?” For all products used, respondents were asked, “The last time you stopped in the past 12 months because you were trying to quit, how long did you stop for?”

### Methods used to quit

At each wave, current smokers received this introduction, “Thinking back to the last time you tried to quit cigarettes in the past 12 months” followed by the three separate queries, “did you use an e-cigarette to help you quit?”; “did you use a nicotine patch, gum, inhaler, nasal spray, lozenge or pill?”; “did you use Chantix, varenicline, Wellbutrin, Zyban, or bupropion?”.

Our hierarchical analytic structure starts with comparing those who used an e-cigarette on the LQA (n = 566) with all “others” (n = 1877). About 20% (n = 116) of those who used an e-cigarette on the LQA also used a pharmaceutical aid; however, with this group separated out, the propensity models did not converge due to the small sample size. Additional sensitivity analyses showed no group differences with and without this group included. Therefore, categories were combined into a single e-cigarette use group. In the next stage of the approach, we consider two sub-groups among the “others”: those who did not use any product on the LQA (n = 1435), and those who used a pharmaceutical aid (but no e-cigarette) (n = 442). We define a daily e-cigarette group as those who used e-cigarettes for the LQA and were daily e-cigarette users at W2 (n = 162) and compare them to non-e-cigarette users on both LQA and W2 (n = 1598). This daily e-cigarette group is missing respondents who were daily e-cigarette users at LQA but discontinued daily use by W2. Abstinence duration at an earlier time-point is a strong predictor of abstinence 1 year later [20]. To control for this potential confounding, we stratify by W2 abstinence duration status (quit 30+ days or not).

### Study covariates

Prior to analysis, we identified candidate W1 variables that might be related to use of cessation aids [21], or e-cigarettes [22], or study outcomes. Briefly, these included sociodemographic variables, cigarette smoking history, nicotine dependence [23], quitting history, timing of LQA, relative perceived harm (cigarettes, e-cigarettes), social variables (smoke-free home, exposure to other smokers), and other covariates (symptoms for externalizing and internalizing mental health problems [24] and smoking-related health conditions). Measurement details for these are in Supplement 1 in S1 Material and the univariate distribution by method used to quit is in Supplement 2 in S1 Material.

### Statistical analyses

For population analyses, we used all-waves weights from the PATH Study Restricted Use Files to obtain estimates that are representative of the civilian, non-institutionalized US population at the time of W1 while considering censoring across waves. For unadjusted population estimates for each comparison group, weighted percentages and Wilson confidence limits (CLs) for proportions were calculated, using balanced repeated replication with Fay adjustment (ρ = 0.3) [25] in SAS (version 9.4).

To model abstinence by method used to quit, we use 1:1 Propensity Score Matching (PSM) [19, 26]. The propensity score (PS) is the probability of group membership conditional on observed covariates, estimated from logistic regression. For each analysis, we assumed a missing-at-random pattern for covariates as all covariates were measured prior to the assessment of the exposure and/or outcome. Missing data was imputed using the Mice package in R (R Foundation for Statistical Computing, Vienna, Austria, version 3.5.1). Given the correlation between covariates, for each PS model we implemented variable selection using the LASSO and the Akaike Information Criterion [27, 28]. For inference, we used multiple bootstrap replications with the number determined by a jackknife quality estimate <0.1 [29]. This resulted in 1500 models for the binary exposure comparisons of e-cigarette vs no e-cigarette use as well as no product use, and 2000 models for comparisons of e-cigarettes with pharmaceutical aids as well as for the stratified daily e-cigarette use comparisons. Each PS model had a different imputed dataset with a different set of covariates.

Using the PS, we matched each exposed respondent to the unexposed control respondent with the nearest PS value using the R package MatchIt, with all PS differences required to be within either 0.1 or 0.2 based on the lowest standardized difference averaged across the selected covariates after matching [30]. Supplement 3 in S1 Material shows the improvement from PSM, using kernel density plots for randomly selected bootstrap examples, as well as boxplots of the standardized mean difference between groups across the bootstrap samples, before and after matching. When more than half of bootstrap samples had mean between-group differences with a > 0.1 standard deviation, that covariate was determined to be substantially unbalanced. Associations were estimated by logistic regression, including a group indicator and fixed effects for each matched exposure pair. We report the mean from the distribution of the bootstrap models as the central estimate, with bootstrap percentile confidence intervals. We applied a Bonferroni adjustment for multiple comparisons made for each outcome measure.

### Sensitivity analyses

**Fig 2:**
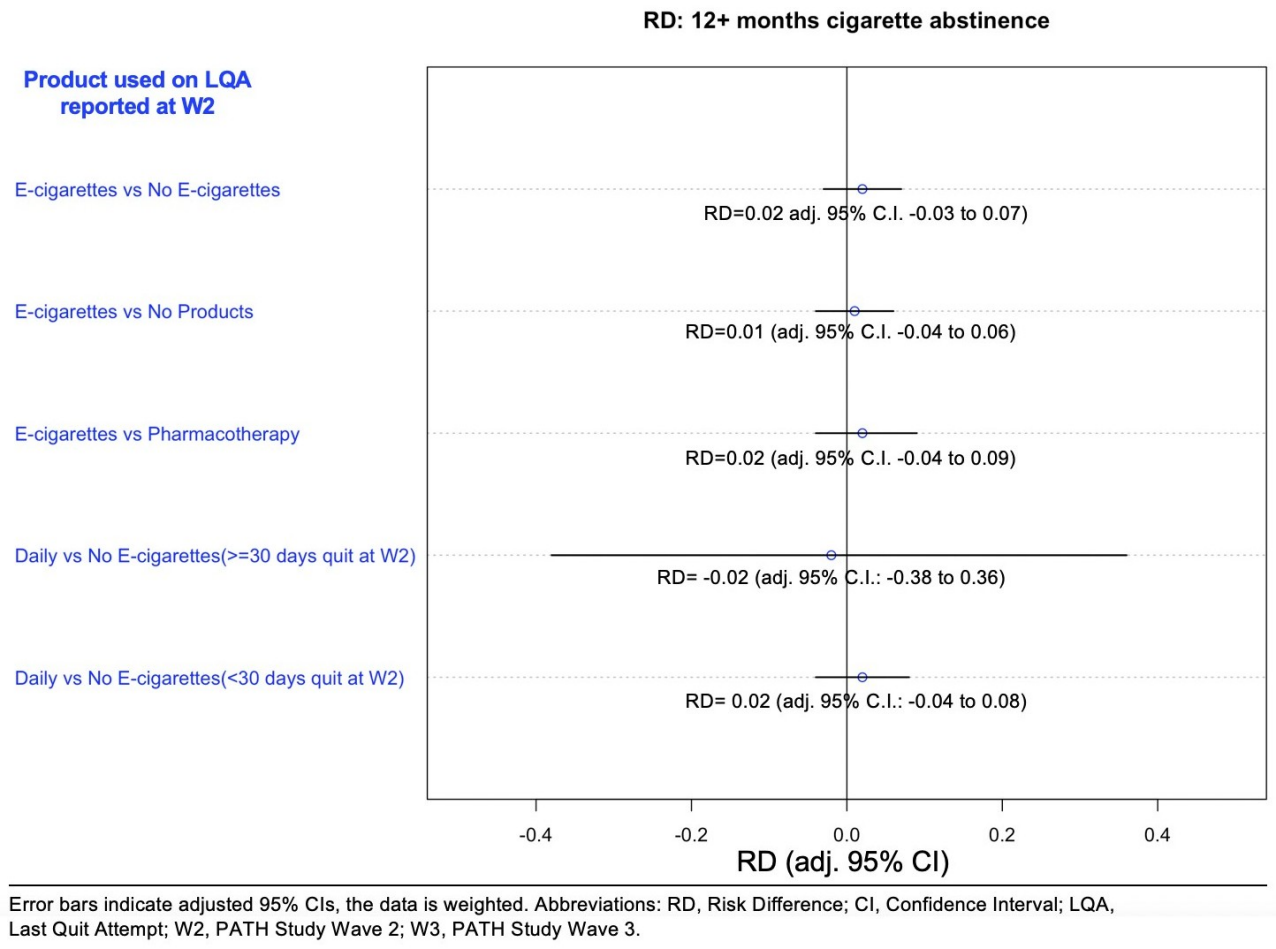
Net risk difference^a^ between matched samples of e-cigarette users and comparison groups in cigarette abstinence for 12+ months at W3. ^a^Risk difference is the difference between the risk of an outcome in the exposed group and the unexposed group. Confidence intervals in this figure are Bonferroni adjusted to account for the 10 different comparisons that were undertaken in Fig 2 and Fig 3. For example, this adjustment means that the CIs for the e-cigarette vs no e-cigarette comparison in Fig 2 changed from -0.02 to 0.05. to a more conservative -0.03 to 0.07.

**Fig 3:**
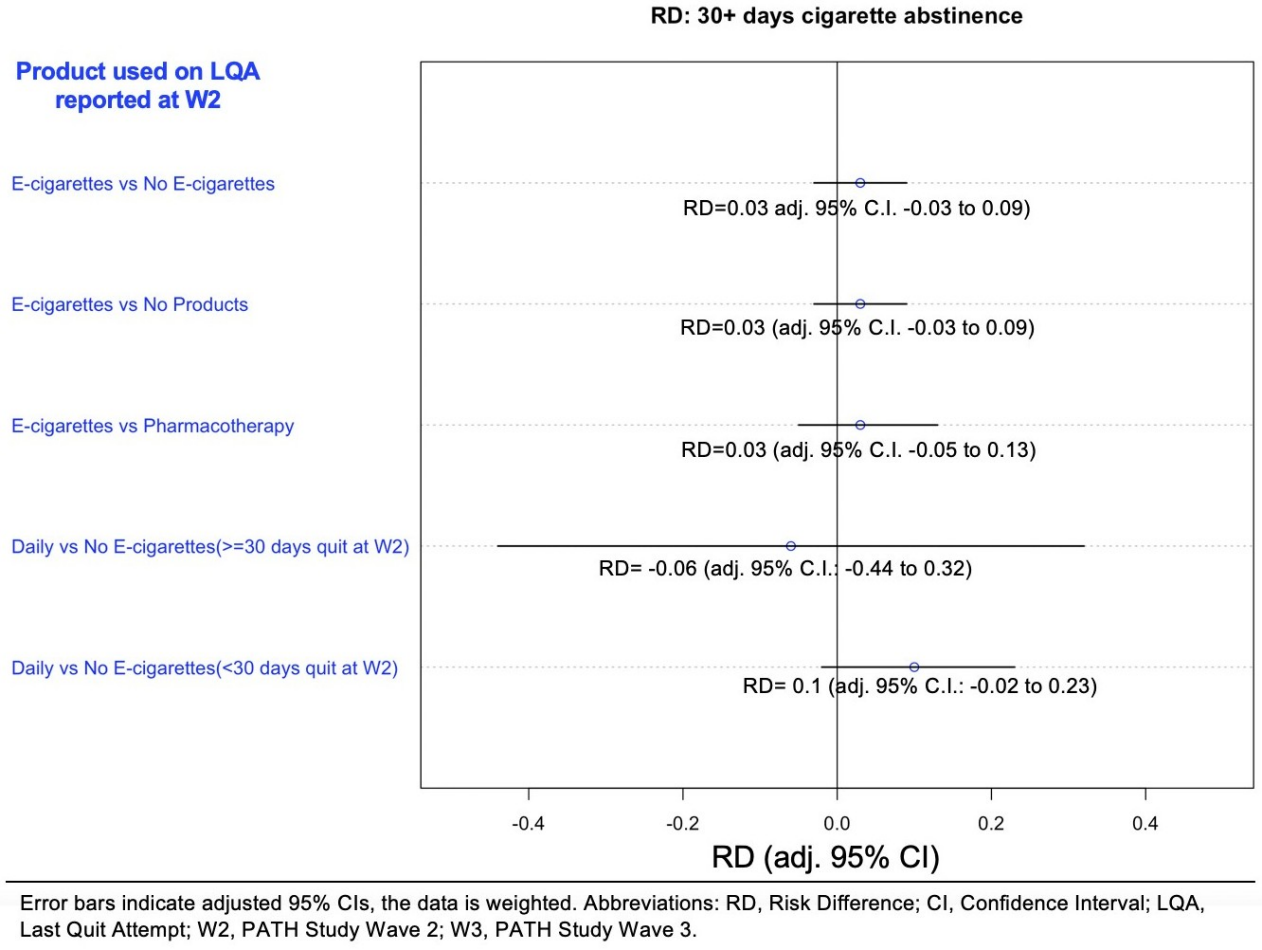
Net risk difference^a^ between matched samples of e-cigarette users and comparison groups in cigarette abstinence for 30+ days at W3. ^a^Risk difference is the difference between the risk of an outcome in the exposed group and the unexposed group. Confidence intervals in this figure are adjusted to account for the 10 different comparisons that were undertaken in Fig 2 and Fig 3. For example, this adjustment meant that the CIs for the e-cigarette vs no e-cigarette comparison in Fig 3 changed from -0.02, 0.07 to a more conservative -0.03 to 0.09.

For each of our main comparisons, we undertook a series of sensitivity analyses using multivariable logistic regression on the full sample (Supplement 4 in S1 Material). The main set of sensitivity analyses for cigarette abstinence repeated the PSM analyses and included 10 comparisons (see Figs 2 and 3 and S4 Supplement in S1 Material). We report timing of LQA across methods used to quit [31] (Supplement 5 in S1 Material), and present the main sensitivity analyses stratified by a binary LQA recall variable (secondary set 1, Supplement 4 in S1 Material, 20 comparisons). For our hypothesis on daily e-cigarette use, the way that the PATH Study estimates daily e-cigarette use may induce possible survivorship bias, so we conducted an additional set of sensitivity analyses. While others have restricted the LQA to within a month of the survey [31], our approach is to use two counterfactual scenarios to explore the sensitivity of our findings. In one scenario, we assume that daily e-cigarette users at W2 represent half of those who were daily on the LQA (supported by a recent randomized trial [10]), thus, in our study, all who reported e-cigarette use on the LQA are assumed to have started as daily users. The second scenario assumes that there were no other daily e-cigarettes users apart from those identified at Wave 2.

## Results

### Study population

Of the 9021 daily cigarette smokers identified at W1, 2770 of these reported a recent quit attempt at W2 and 2443 completed W3 (Fig 1). Some 23.5% reported use of e-cigarettes as method to quit smoking on LQA. A pharmaceutical aid but not an e-cigarette was used by 19.3%, of whom 60.5% used only NRT, 17% used only prescription medications and 22.5% used both. The no product group was 57.2%. Many covariates differed between these groups (Supplement 2 in S1 Material). Comparing the e-cigarette and pharmaceutical groups over the 2000 matching models, there were 10 covariates that were substantially unbalanced in the population data with the top three being age, pack years and relative perceived harm of e-cigarettes (for the full list see Supplement 3 in S1 Material). Comparing e-cigarettes and no product groups over 1500 matching models, there were 13 covariates that were substantially unbalanced in the population data with the top three being relative perceived harm of e-cigarettes, nicotine dependence and race.

### Abstinence outcomes by product (unadjusted)

At W3 follow-up, there were no differences in the proportions who were abstinent from cigarettes for 12+ months (~10%) or for 30+ days at W3 (~16%), nor was there a difference in current cigarette smoking (~80–85%) whether the method used to quit was e-cigarettes, pharmaceutical aids, or neither product (Table 1).

**Table 1:** Weighted U.S. population estimates^a^ of abstinence outcomes at wave 3 by product used on last quit attempt among PATH Study W1 daily smokers who made a quit attempt prior to W2^b^.

	Cessation Outcome by E-Cigarette Use on Last Quit Attempt Reported at W2			
	E-cigarette Used for LQA^d^	No E-cigarette Used for LQA		
		No Pharmaceutical on LQA	Any Pharmaceutical Aid for LQA^f^	Overall
	n = 566^e^	n = 1435	n = 442	n = 1877
	Weighted % (95% CL)	Weighted % (95% CL)	Weighted % (95% CL)	Weighted % (95% CL)
**Abstinence Outcome**				
**W3 abstinent all tobacco**^c^^,^^d^ **(incl. e-cigarettes) 12+ mo**	3.2 (2.0–5.1)	7.7 (6.3–9.5)	5.8 (3.7–8.8)	7.2 (6.0–8.7)
**W3 cigarette abstinent 12+ mo**^d^	9.6 (7.1–13.0)	10.2 (8.5–12.1)	7.4 (5.1–10.7)	9.5 (8.1–11.1)
**W3 cigarette abstinent 30+ days**	17.3 (14.2–21.1)	16.9 (14.7–19.3)	13.8 (10.8–17.5)	16.1 (14.2–18.2)
**W3 current cigarette smoker**	79.7 (75.8–83.2)	80.7 (78.1–83.1)	85.0 (81.0–88.3)	81.8 (79.6–83.8)

*Abbreviations*: CL, Wilson Confidence Limit; LQA, last quit attempt; W1, PATH Study Wave 1; W2, PATH Study Wave 2; W3, PATH Study Wave 3

^a^ Weighted U.S. population estimates derived using the balanced repeated replication method with Fay adjustment.^19^ Confidence Limits are not Bonferroni adjusted.

^b^ Sample includes PATH Study W1 daily smokers (U.S. adults aged 18+) who reported a quit attempt in the past year at W2 and completed W3 (n = 2443).

^c^ All tobacco includes cigarettes, e-cigarettes, cigars (traditional, cigarillo & filtered), pipes, hookah, snus or other smokeless products.

^d^ For the e-cigarette group, the difference between cigarette abstinence and tobacco abstinence (9.6–3.2 = 6.4%), was mainly made up by continued e-cigarette use (5%).

^e^ The e-cigarette group (n = 566) includes e-cigarette users who also used a pharmaceutical aid (n = 116). The comparison group was stratified between those who did use the product (other than e-cigarettes) and those who did not (no product group), as a sensitivity analysis. No differences between these groups were found

^f^ The pharmaceutical aid group does not include 116 users who also used an e-cigarette

For the outcome of abstinence from all tobacco products including e-cigarettes, those who used e-cigarettes for the LQA were less likely to be abstinent for 12+ months, compared to those who quit without e-cigarettes (3.2%; 95% CL, 2.0–5.1 vs 7.2%; 95% CL, 6.0–8.7) (Table 1). However, for the e-cigarette group, an additional 5.0% (95% CL, 3.3–7.5%) were not tobacco abstinent only because they used e-cigarettes at W3 (i.e. 5.0% of 9.6% = 52% of e-cigarette group abstinent from cigarettes were still e-cigarette users).

### Abstinence outcomes at W3 by daily use of e-cigarettes at W2 (unadjusted)

Daily e-cigarette users who were 30+ days abstinent at W2 were not different from the no e-cigarette control at W3 for 12+ month cigarette abstinence (~45%), 30+ days cigarette abstinence (~60%), or current smoking (~35%) (Table 2). None in the daily e-cigarette use group (n = 56) and 45% (95% CL, 36.8–53.5%) of the no e-cigarette group (n = 162) were abstinent from all tobacco for 12+ months at W3.

**Table 2:** Weighted U.S. population estimates^a^ of abstinence at wave 3 for e-cigarette use on LQA and daily use at wave 2 compared to no e-cigarette use, stratified by abstinence level at W2: The PATH Study sample^b^.

	W2 Cigarette Abstinence Status for Daily E-cigarette Group vs No E-Cigarette Group			
	Cigarette Abstinent 30+ Days at W2		Not Cigarette Abstinent 30+ Days at W2	
	E-cig to quit and daily at W2, n = 56	No e-cig at LQA or W2, n = 162	E-cig to quit and daily at W2, n = 106	No e-cig at LQA or W2, n = 1169
	Weighted % (95% CL)	Weighted % (95% CL)	Weighted % (95% CL)	Weighted % (95% CL)
**Wave 3 Study Outcome**				
** 12+ mo abstinent tobacco**^c^ **(including e-cigarettes)**	0.0 (0.0)	45.0 (36.8–53.5)	0.0 (0.0)	1.7 (1.0–3.0)
** Cigarette abstinent 12+ months**	43.7 (31.6–56.7)	52.9 (44.1–61.5)	*	1.8 (1.1–3.0)
** Cigarette abstinent 30+ days**	55.7 (42.7–67.9)	64.1 (55.0–72.3)	16.9 (11.0–25.2)	8.6 (6.8–10.7)
** Current cigarette smoker**	38.1 (26.5–51.2)	34.9 (26.8–44.0)	77.3 (68.5–84.3)	89.6 (87.3–91.5)

*Abbreviations*: CL, Wilson Confidence Limit; e-cig, e-cigarette; LQA, last quit attempt; W1, PATH Study Wave 1; W2, PATH Study Wave 2; W3, PATH Study Wave 3

^a^ Weighted U.S. population estimates derived using the balanced repeated replication method with Fay adjustment [19].. Confidence limits are not Bonferroni adjusted.

^b^ This analysis is limited to the subsample who used an e-cigarette to quit and were daily users at W2 (6.7% of sample) and the control group who did not use an e-cigarette to quit and were not using an e-cigarette at W2. Neither group excludes those who used a pharmaceutical aid to quit.

^c^ All tobacco includes cigarettes, e-cigarettes, cigars (traditional, cigarillo & filtered), pipes, hookah, snus or other smokeless products

* Estimate suppressed because it has low statistical precision. It is based on a denominator sample size of less than 50, or the coefficient of variation of the estimate or its complement is larger than 30%

Among daily e-cigarette users who were less than 30 days abstinent at W2, very few were cigarette abstinent for 12+ months at W3. A higher proportion of the daily e-cigarette group (n = 106) compared to no e-cigarette group (n = 1169) were abstinent for 30+ days at W3 (16.9%; 95% CL,11.0–25.2 vs 8.6%; 95% CL, 6.8–10.7).

### PS adjusted net risk difference in 12+ month cigarette abstinence at W3

The PS-matching achieved excellent covariate balance between each of the study comparison groups, as seen from the kernel density plots and the covariate balance grids (Supplement 3 in S1 Material). There was no net difference in the 12+ months cigarette abstinence rates (Fig 2) between the e-cigarette user group and the matched no e-cigarette controls (Risk Difference (RD), 0.02, [adj. 95% CI, -0.03 to 0.07]), the matched no product controls (RD, 0.01 [adj. 95% CI, -0.04 to 0.06]), or the matched pharmaceutical aid control (RD, 0.02 [adj. 95% CI,-0.04 to 0.09]). Similarly, there was no net risk difference between the W2 daily e-cigarette group and the matched no e-cigarette control, regardless of whether they were 30+ days abstinent at W2 (RD, -0.02 [adj. 95% CI, -0.38 to 0.36]) or not (RD, 0.02 [adj. 95% CI, -0.04 to 0.08]).

### PS adjusted net risk difference in 30+ days cigarette abstinence at W3

There was no net risk difference in 30+ days cigarette abstinence at W3 between the e-cigarette group and either the no e-cigarette control (RD, 0.03 [adj. 95% CI, -0.03 to 0.09]), the no product control (RD, 0.03 [adj. 95% CI, -0.03 to 0.09]) or the pharmaceutical aid control (RD, 0.03 [adj. 95% CI, -0.05 to 0.13]) (Fig 3). Among the daily e-cigarette use subgroup who were abstinent for 30+ days at W2, there was no risk difference for daily e-cigarette users compared to the no e-cigarette control (RD, -0.06 [adj. 95% CI, -0.44 to 0.32]). Similarly, no difference was observed for those who were not 30+ days cigarette abstinent at W2 (RD, 0.10 [adj. 95% CI, -0.02 to 0.23]).

### PS adjusted net risk difference for 12+ month all tobacco abstinence at W3

**Fig 4:**
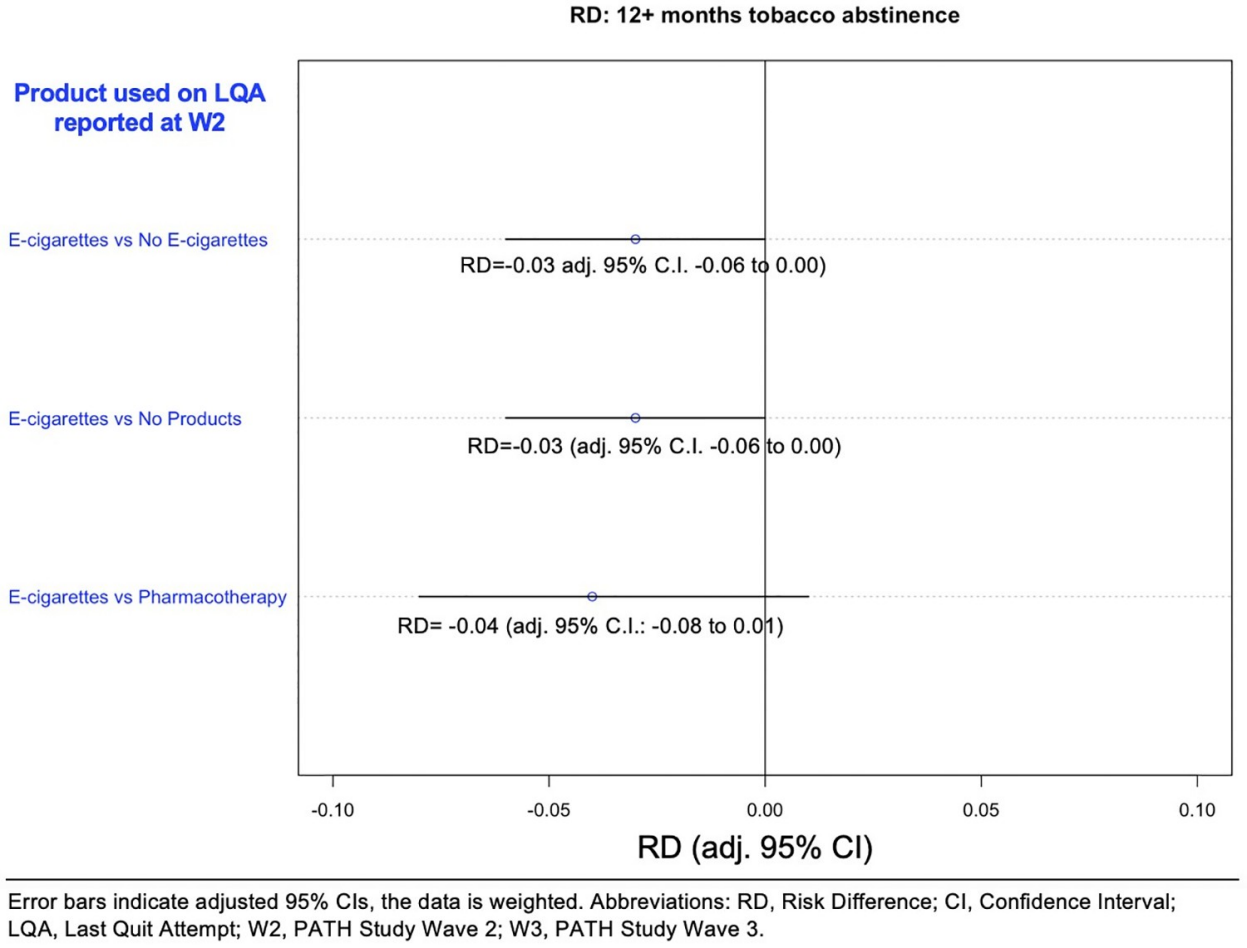
Net risk difference^a^ between matched samples of e-cigarette users and comparison groups in tobacco abstinence (including e-cigarettes) for 12+ months at W3. ^a^Risk difference is the difference between the risk of an outcome in the exposed group and the unexposed group. Confidence intervals in this figure are Bonferroni adjusted to account for the 3 different comparisons that were undertaken in Fig 4. For example, this adjustment meant that the CIs for the e-cigarette vs no e-cigarette comparison in Fig 4 changed from -0.05 to -0.004 to a more conservative -0.06 to 0.00.

The proportion with 12+ months abstinence from all tobacco (including e-cigarettes) was 3 percentage points lower in the e-cigarette group than in either the matched no e-cigarette control (RD, -0.03 [adj. 95% CI, -0.06 to 0.00]) or the matched no product control (RD, -0.03, [adj. 95% CI, -0.06 to 0.00]), although the upper CL for both included zero. A similar lower 12+ month tobacco abstinence was observed between e-cigarette users and matched pharmaceutical cessation aid control (RD, -0.04 [adj. 95% CI, -0.08 to 0.01]) although the upper confidence interval for this estimate crossed zero (Fig 4).

### Sensitivity analyses (supplement 4 in S1 Material)

The first set of sensitivity analyses (Supplement 4 in S1 Material) assessed the main study comparisons using logistic regression among all US daily smokers, adjusted for the same set of confounders as the PSM analyses. Results were consistent with the PSM findings, with the exception that daily e-cigarette users who had not quit for 30+ days at W2 were identified as more likely to be abstinent for 30+ days at W3 than the no e-cigarette controls. The secondary set 1 of sensitivity analyses identified that the daily e-cigarette users who were less than 30+ days quit at W2 but who were quit for 30+ days at W3 had an LQA earlier in the W1- W2 time period, and, hence, had relapsed at W2. The secondary set 2 tested two hypothetical assumptions of who were daily e-cigarette users on the LQA among those who had quit in the month prior to W2. Neither assumption tested suggested that daily e-cigarette use for the LQA was associated with 12+ month cigarette cessation at W3.

## Discussion

In this large representative sample of US daily cigarette smokers with a recent quit attempt, we found no evidence to support our first hypothesis, that ENDS would be associated with increased abstinence duration from either cigarettes or any tobacco. Twelve-month cigarette abstinence was ~10% and comparable across all methods used to quit, and the finding was robust to different sensitivity analyses. Over half of those who used e-cigarettes to try to quit were still using e-cigarettes at W3 follow-up.

Similarly, our results did not support our second hypothesis, that daily use of ENDS at Wave 2 would be associated with increased cigarette abstinence. Recent evidence has suggested that e-cigarettes might need to be used daily to increase cessation rates among daily smokers [32] The PATH Study did not ask about frequency of use of e-cigarettes on the last quit attempt. Rather, current daily use was available for the time of the interview, which, in this sample was a median of 138 days (95% CL, 129–148) from LQA (see Supplement 5 in S1 Material). In a recent randomized trial of e-cigarettes used for cessation [11], 53% of the e-cigarette group still used e-cigarettes daily at the 4-week study time-point. Further, evidence from NRT studies suggests that discontinuation of a cessation product is often associated with relapse to smoking, although the causal direction may go either way [33]. Because length of cessation at an intermediate time-point from a quit attempt is a major predictor of long-term success [20], for the daily e-cigarette use analysis, we stratified use at W2 by 30+ day cigarette abstinence at W2 and observed no differences in 12+ month cigarette abstinence rates.

We conducted additional sensitivity analyses by limiting the analysis to those who reported cigarette quit attempts within one month of the W2 interview [34] and tested two hypothetical scenarios of who were daily e-cigarette users on the LQA. Sample sizes were small but these sensitivity analyses did not suggest that daily e-cigarette use made a difference in W3 cigarette abstinence rates.

There were mixed results on whether daily e-cigarette use at W2 was associated with 30+ days abstinence at W3. Both the unadjusted population estimates and the sensitivity analyses suggested that there might be such an association (the secondary sensitivity analysis indicated that such an association was limited to those who had relapsed by W2), but no association was present in the PSM analysis. The PSM result is similar to that of a recent non-representative observational study [35] that dual use of cigarettes and e-cigarettes is not associated with improved long-term abstinence rates. If an association exists, the mechanism is not clear and is likely complex and beyond the scope of this paper.

The results for e-cigarettes as a method to quit smoking in this population-based cohort differ from the conclusion in a recent randomized controlled trial [11]. In the trial, the e-cigarette group included 4 weeks of counseling and abstinence at one year was higher than in the NRT comparison group (18% vs 10%). Randomized trials are well suited for demonstrating efficacy, but they do not always translate to the general population [8, 36]. Reasons for this include that the optimal cessation aid package tested in trials is rarely what is implemented at the population level [37]. Further, the selection criteria for randomized trials may limit generalizability to the population.^36^ In addition, different measurement methods may result in different outcomes between randomized trials and population-based studies. Previous studies of NRT have also found clear evidence of efficacy in trials [38] but these have not translated consistently to the general population level for effectiveness of NRT [37]. Understanding what leads to the differences between these types of studies is important to optimize the public health potential and ways to deliver these interventions. The e-cigarette market is highly diverse and is evolving. As such, the findings here may not extend to newer, more efficient e-cigarette designs, some of which may deliver nicotine as effectively as cigarettes [39]. Our data show that the majority of smokers try to quit without use of an e-cigarette or pharmaceutical aid, and this is as effective as any other method. However, only 10% were able to stay abstinent for 12+ months from any method used to quit, indicating major room for improvement.

A strength of this study is that it is a large representative sample of the US population that is being followed longitudinally. As such, it provides evidence of the current real-world benefit of e-cigarettes and FDA-approved pharmacotherapy as they were used at the time by US daily smokers. We used 3 waves of data and so were able to identify daily smokers at W1, assess method used to quit at W2, and assess abstinence outcomes at W3. This study used PS matching to obtain an excellent balance across groups on the many covariates associated with methods used to quit. This addresses a common criticism of causal inference from observational studies [18, 40]. In addition, the results were robust to a variety of sensitivity analyses as demonstrated in the Supplemental Tables in S1 Material.

There are several limitations. The PATH Study does not assess the frequency of use of e-cigarettes at the LQA nor does it track the outcomes associated with a particular attempt to quit smoking. A limitation of any observational study is unmeasured confounding that is uncorrelated with the covariates included in the PS model. Quit attempt data are recalled retrospectively and abstinence is self-reported; however, in the past, population studies of smoking behavior have been shown to be reasonably valid [41, 42]. Moreover, the small sample size of the 20% of e-cigarette users who also use a pharmaceutical aid did not allow for examining an e-cigarette only group compared to a pharmaceutical aid only because propensity score models did not converge; however, sensitivity analyses found no difference between these groups, suggesting this issue did not have a major impact on the results. The PATH Study also queried quit attempts that generally occurred between 2014 and 2015 and cessation results with products and device types used at that time might not generalize to products available more recently.

## Conclusion

In this representative sample of the US population, those who used e-cigarettes as a method to quit cigarette smoking had similar 12+ months cigarette abstinence rates, assessed 1–2 years after the quit attempt, compared to those who used FDA-approved cessation aids or no product at all. However, over half those who used e-cigarettes in their quit attempt still used e-cigarettes a year later. Further studies can identify under what conditions, if any, e-cigarettes might assist in the public health effort to increase smoking cessation at the population level. It is important to continue to follow this population sample as e-cigarettes have evolved rapidly, and the process of cigarette cessation can take many years before the long-term effects of a potential new method to quit become clear.

## Supporting information

S1 Material (DOCX)
