# Supplementary material for: Role of e-cigarettes and pharmacotherapy during attempts to quit cigarette smoking: The PATH Study 2013-16
Source: PLoS One. 2020 Sep 2;15(9):e0237938. doi: 10.1371/journal.pone.0237938 (PMC7467279; doi:10.1371/journal.pone.0237938)
Supplement: S1 Material — (DOCX) [file pone.0237938.s001.docx]

**Supplementary Material**

Role of e-cigarettes and pharmacotherapy during attempts to quit cigarette smoking: the PATH Study 2013-16

Table of Contents

[Supplement 1. Measurement Detail for Pre-identified Study Covariates in PATH Study (with variable names) 2](#_Toc32500142)

[Supplement 2. Univariate Distribution of Study Covariates by Product Category 4](#_Toc32500143)

[Supplement 3. Improvement in Covariate Balance with Propensity Score Matching: Comparability of Groups (Kernel Density Plots and Average Covariate Balance Grid across all Bootstrap runs) 7](#_Toc32500144)

[Supplement 4. Sensitivity Analyses for PSM models 15](#_Toc32500145)

[a. Main Sensitivity Analyses 15](#_Toc32500146)

[Findings from the Main Set of Sensitivity Analyses 16](#_Toc32500147)

[b. Secondary Sensitivity Analyses 1: Stratification on Timing of LQA 16](#_Toc32500148)

[Findings from Secondary Sensitivity Analyses 1 17](#_Toc32500149)

[c. Secondary Sensitivity Analyses 2: Hypotheticals among E-cigarette users with LQA <1 month prior to W2 18](#_Toc32500150)

[Unadjusted Population Estimates for Long-Term Cigarette Abstinence at W3 18](#_Toc32500151)

[Findings from Secondary Sensitivity Analyses 2 19](#_Toc32500152)

[Supplement 5. Timing of the Start of the Last Quit Attempt prior to Wave 2 by Product Used 20](#_Toc32500153)

# Supplement 1. Measurement Detail for Pre-identified Study Covariates in PATH Study (with variable names)

#### Sociodemographics

We use standard derived variables for **age, sex, education, race-ethnicity, and poverty level** (R01R_A_AGE_IMP, R01R_A_SEX_IMP, R01R_A_EDUC_IMP, R01R_A_ETHRACECAT5_IMP, R01R_POVERTYPERCENT). Poverty level categorizes income as a percentage of the Federal Poverty Level (FPL). Note, missing data on age, gender, race, Hispanic ethnicity, adult education were imputed using existing information from other PATH Study sources as described in the PATH Study Restricted Use Files User Guide (United States Department of Health and Human Services 2018, https://doi.org/10.3886/ICPSR36231.v19).

#### Nicotine dependence

(R01_AN0025, R01_AN0030, R01_AN0065, R01_AN0035, R01_AN0045, R01_AN0085, R01_AN0090, R01_AN0060, R01_AN0095, R01_AN0100, R01_AN0055, R01_AN0050, R01_AN0070, R01_AN0075, R01_AN0080). Variables are combined to derive variable R01R_A_DPNDSCL_NOMISS by calculating the mean of the non-missing scores. Nicotine dependence items take the form of a series of statements on emotional and physical responses to nicotine products (e.g. “I frequently crave {product}”, “I usually want to {use product} right after I wake up”, “I [would ] feel alone without my {product}”). Respondents are asked to rate their level of agreement with each statement on a 5-point scale, where 1=”Not true of me at all” and 5=”Extremely true of me”. Respondents can also answer “don’t know” or refuse to answer the question; these are treated as missing responses. Responses are rescaled to a 3-point scale, where 1 (not at all)=0, 2 or 3 = 50 and 4 or 5 =100, summed and divided by the number of non-missing values.

#### Cigarette consumption

(R01R_A_PERDAY_EDY_CIGS). Average number of cigarettes smoked each day. Responses could be reported as cigarettes or packs. Respondents who reported smoking more than 5 packs/day were considered to be errors and were recoded as number of cigarettes.

#### Length of LQA reported at W1

(R01R_A_DURQUIT) – Length of LQA (in days) in the past 12 months reported at Wave 1. Note this was a failed quit attempt in this sample.

#### Timing of LQA

(R02R_A_DAYSUNTILQA_CIGS) This was calculated as the date of the most recent quit attempt reported in W2 started minus the date of the W1 survey. If respondents reported a start date prior to the W1 survey, the number will be negative. As all respondents were daily smokers at W1, we recoded the LQA to occur just after W1. In Supplement 6, this variable is reported as ‘Timing of LQA from W2’ which is the way that most people think of this variable. This is the complement to the ‘Timing of LQA after W1’ with approximately 1 year between W1 and W2.

#### Smoke-free home

(R01_AR1045). Those who responded “It is not allowed anywhere or at any time inside my home” to the question “For tobacco products that are burned, such as cigarettes, cigars, pipes or hookah, which statement best describes the rules about smoking a tobacco product inside your home?” were scored as having a smoke-free home; all others, including those who refused to answer or answered “Don’t know” were scored as not having a smoke-free home.

#### Perceived harm of cigarettes

(R01_AC9050). Respondents were asked “How harmful do you think cigarettes are to health?“ and could reply on a 5-point scale from 1 (not at all harmful) to 5 (extremely harmful).

#### Relative perceived harm of e-cigarettes

(R01_AE1099). Respondents were asked “Is using e-cigarettes less harmful, about the same, or more harmful than smoking cigarettes?” and could reply on a 3-point scale, where 1=Less harmful, 2=About the same and 3=More harmful.

#### Exposure to other smokers

(R01_AX0068) Respondents were asked “During the past seven days, about how many hours were you around others who were smoking [, whether or not you were smoking yourself]? Include time in your home, in a car, at work, or outdoors.”

#### Pack years of smoking

(R01R_A_PACKYEARS_CIGS). Calculated by multiplying the number of pack smoked per day by the number of years the respondent smoked regularly.

#### Age started smoking fairly regularly

(R01R_A_AGEREG_CIGS)

#### Interest in quitting cigarettes

(R01_AN0230) on a scale of 1-10 where 1=Not at all interested and 10=Extremely interested.

#### Self-efficacy about quitting

(R01_AN0245) “If you did try to quit {product} altogether in the next 6 months, how likely do you think you would be to succeed?” on a 4-point scale from 1=Not at all likely and 4=Very likely.

#### Smoking-related health diagnoses

Respondents were asked if they had ever been told by a doctor or health professional that they had any of a list of diseases.We included the following more common conditions among respondents in this sample:

- High blood pressure (R01_AX0111_01)
- High cholesterol (R01_AX0111_02)
- Other heart condition (R01_AX0111_06)
- Chronic bronchitis (R01_AX0119_02)
- Asthma (R01_AX0119_04)
- Cancer: (R01_AX0144)

#### Disorder symptoms for externalizing mental health problems

(R01R_A_GAINEXTSCREEN_PY). Respondents were asked the last time they had experienced any of 7 externalizing (e.g., had a hard time paying attention or listening to instructions at school, work or home, bullied or started physical fights). The number of reports of experiencing such symptoms in the past month or the past 2-12 months was summed and coded into a 3-level severity indicator, with those reporting 0 or 1 symptoms scored as Low, 2-3 symptoms scored as Moderate and 4 or more scored as High.

#### Disorder symptoms for internalizing mental health problems

(R01R_A_GAININTSCREEN_PY) Respondents were asked the last time they experienced any of the following internalizing disorder symptoms: feeling very trapped, lonely, sad, blue, depressed, or hopeless about the future, feeling very anxious, nervous, tense, scared, panicked, or like something bad was going to happen, had sleep problems. The number of reports of experiencing such symptoms in the past month or the past 2-12 months was summed and coded into a 3-level severity indicator, with those reporting 0 or 1 symptoms scored as Low, 2-3 symptoms scored as Moderate, and 4 or more scored as High.

#### Health insurance status

(R02_AM0026_01 to R02_AM0026_08). Respondents who reported currently being covered by at least one type of health insurance, including insurance purchased directly or through an employer or union, Medicare, Medicaid, VA, TRICARE or other military health care and Indian Health Insurance, were scored as having insurance coverage. Missing data on all of these variables were coded to “did not have insurance.”

# Supplement 2. Univariate Distribution of Study Covariates by Product Category

In eTable 1, the column N is unweighted but the estimates (%, 95% CL) are weighted. The weighted percent (**wtd %**) is the population in row category who used the product in the column header (for example, among thosed aged 18-34, 28.4% used e-cigs on LQA, 11.1% used other products, etc.). The different columns in this table reflect columns in Tables 1 and 2 of the main paper. Those who used e-cigarettes on LQA are from column 2 of Table 1, those who used other products on LQA are from column 4 of Table 1 and those who did not use e-cigarettes on LQA are from column 5 of Table 1. Those who used e-cigarettes on LQA and daily at W2 are from columns 2 and 4 in main paper Table 2. Those who did not use e-cigarettes on LQA or W2 are those from columns 3 and 5 of main paper Table 2.

| eTable 1. Univariate Distribution of Study Covariates by Product Category | | | | | | | | | | | | | | | | | | | | | |
| --- | --- | --- | --- | --- | --- | --- | --- | --- | --- | --- | --- | --- | --- | --- | --- | --- | --- | --- | --- | --- | --- |
| **Parameter (Variable name)** | | Used e-cigarettes on LQA (n=566) | | | | Any Pharmaceutical Aid for LQA (n=442) | | | | Did not use e-cigarettes on LQA (n=1877) | | | | Used e-cigarettes on LQA and daily at W2 (n=162) | | | | No use of e-cigarettes on LQA or W2 (n=1331) | | | |
|  | | N | wtd % | 95% CL | | N | wtd % | 95% CL | | N | wtd % | 95% CL | | N | wtd % | 95% CL | | N | wtd % | 95% CL | |
| **Age** | 18-34 | 296 | 28.4 | 25.2 | 31.8 | 111 | 11.1 | 9.1 | 13.3 | 802 | 71.6 | 68.2 | 74.8 | 95 | 9.0 | 7.2 | 11.2 | 578 | 51.0 | 47.0 | 55.0 |
| (R01R_A_AGE_IMP) | 35-59 | 236 | 22.4 | 19.6 | 25.4 | 255 | 24.4 | 21.5 | 27.6 | 832 | 77.6 | 74.6 | 80.4 | 59 | 5.6 | 4.4 | 7.2 | 579 | 53.7 | 50.2 | 57.3 |
|  | 60+ | 34 | 12.9 | 9.2 | 17.7 | 76 | 26.0 | 21.1 | 31.6 | 243 | 87.1 | 82.3 | 90.8 | ⱡ | | | | 174 | 60.4 | 54.6 | 66.0 |
| **Sex** | 1 = Male | 249 | 21.2 | 18.3 | 24.3 | 203 | 18.2 | 15.4 | 21.3 | 931 | 78.8 | 75.7 | 81.7 | 93 | 7.5 | 6.0 | 9.3 | 638 | 54.2 | 50.8 | 57.7 |
| (R01R_A_SEX_IMP) | 2 = Female | 317 | 26.2 | 23.8 | 28.7 | 239 | 20.6 | 18.1 | 23.3 | 946 | 73.8 | 71.3 | 76.2 | 69 | 5.7 | 4.5 | 7.2 | 693 | 52.7 | 49.8 | 55.5 |
| **Education** | Less than high school | 119 | 17.6 | 14.9 | 20.6 | 125 | 20.2 | 16.4 | 24.6 | 555 | 82.4 | 79.4 | 85.1 | 33 | 5.1 | 3.5 | 7.4 | 334 | 54.0 | 49.6 | 58.3 |
| (R01R_A_EDUC_IMP) | High school graduate | 144 | 24.0 | 19.9 | 28.5 | 101 | 17.8 | 14.2 | 22.1 | 477 | 76.0 | 71.5 | 80.1 | 40 | 7.0 | 5.1 | 9.5 | 373 | 54.7 | 50.5 | 58.8 |
|  | At least some college | 303 | 26.6 | 23.8 | 29.5 | 216 | 19.7 | 17.2 | 22.5 | 845 | 73.4 | 70.5 | 76.2 | 89 | 7.3 | 5.8 | 9.2 | 624 | 52.6 | 49.2 | 55.9 |
| **Race/ethnicity** | Hispanic | 45 | 15.4 | 11.5 | 20.4 | 36 | 12.6 | 8.8 | 17.6 | 237 | 84.6 | 79.6 | 88.5 | ⱡ | | | | 169 | 59.9 | 52.6 | 66.8 |
| (R01R_A_ETHRACECAT5_IMP) | Non-Hispanic Asian/other | 36 | 18.7 | 12.6 | 26.8 | 29 | 15.3 | 9.4 | 24.0 | 140 | 81.3 | 73.2 | 87.4 | ⱡ | | | | 94 | 56.4 | 46.1 | 66.1 |
|  | Non-Hispanic Black | 36 | 10.3 | 6.9 | 15.2 | 58 | 16.1 | 12.5 | 20.5 | 347 | 89.7 | 84.8 | 93.1 | ⱡ | | | | 260 | 66.0 | 59.5 | 71.9 |
|  | Non-Hispanic White | 449 | 27.8 | 25.5 | 30.3 | 319 | 21.3 | 18.7 | 24.2 | 1153 | 72.2 | 69.7 | 74.5 | 136 | 8.3 | 7.0 | 9.8 | 808 | 49.7 | 46.6 | 52.8 |
| **Poverty level** | < 100% FPL | 192 | 19.7 | 17.2 | 22.6 | 141 | 15.7 | 12.9 | 19.0 | 762 | 80.3 | 77.4 | 82.8 | 51 | 5.2 | 4.0 | 6.8 | 524 | 54.8 | 51.2 | 58.3 |
| (R01R_POVERTYPERCENT) | 100-< 200%FPL | 166 | 24.7 | 21.0 | 28.7 | 127 | 20.0 | 16.8 | 23.7 | 495 | 75.3 | 71.3 | 79.0 | 53 | 8.3 | 6.2 | 11.0 | 333 | 48.8 | 43.3 | 54.4 |
|  | 200-< 300% FPL | 54 | 21.3 | 16.3 | 27.2 | 41 | 16.1 | 11.0 | 22.8 | 198 | 78.7 | 72.8 | 83.7 | 12 | 4.7 | 2.7 | 8.1 | 168 | 64.7 | 57.2 | 71.5 |
|  | 300+% FPL | 117 | 31.9 | 26.9 | 37.4 | 89 | 22.5 | 18.2 | 27.5 | 278 | 68.1 | 62.6 | 73.1 | 37 | 9.1 | 6.4 | 12.8 | 206 | 50.6 | 45.2 | 56.0 |
| **Health insurance status** | No | 112 | 24.0 | 20.0 | 28.4 | 41 | 9.6 | 7.0 | 13.1 | 375 | 76.0 | 71.6 | 80.0 | 36 | 7.4 | 5.4 | 10.0 | 260 | 51.5 | 47.0 | 56.0 |
| (R02_AM0026_01-R02_AM0026_08) | Yes | 454 | 23.4 | 21.4 | 25.5 | 401 | 21.7 | 19.1 | 24.4 | 1502 | 76.6 | 74.5 | 78.6 | 126 | 6.5 | 5.4 | 7.8 | 1071 | 54.0 | 51.4 | 56.6 |
| **Disorder symptoms for externalizing mental health problems** | Low | 261 | 21.1 | 18.8 | 23.5 | 245 | 20.7 | 18.5 | 23.0 | 1014 | 78.9 | 76.5 | 81.2 | 73 | 5.9 | 4.6 | 7.4 | 732 | 56.3 | 53.1 | 59.5 |
| (R01R_A_GAINEXTSCREEN_PY) | Moderate | 169 | 28.0 | 24.5 | 31.8 | 101 | 16.5 | 13.2 | 20.5 | 467 | 72.0 | 68.2 | 75.5 | 51 | 8.4 | 6.2 | 11.2 | 331 | 50.1 | 45.9 | 54.4 |
|  | High | 132 | 26.2 | 22.4 | 30.4 | 83 | 17.5 | 13.7 | 22.1 | 353 | 73.8 | 69.6 | 77.6 | 38 | 7.4 | 5.2 | 10.3 | 239 | 49.1 | 44.1 | 54.2 |
| **Disorder symptoms for internalizing mental health problems** | Low | 221 | 20.6 | 18.3 | 23.2 | 205 | 19.6 | 17.2 | 22.3 | 879 | 79.4 | 76.8 | 81.7 | 60 | 5.6 | 4.3 | 7.3 | 628 | 55.7 | 52.1 | 59.3 |
| (R01R_A_GAININTSCREEN_PY) | Moderate | 144 | 23.6 | 20.5 | 27.1 | 109 | 19.2 | 15.3 | 23.7 | 485 | 76.4 | 72.9 | 79.5 | 48 | 7.8 | 5.8 | 10.3 | 362 | 56.8 | 52.2 | 61.2 |
|  | High | 199 | 29.2 | 25.7 | 33.0 | 122 | 18.1 | 14.9 | 21.7 | 489 | 70.8 | 67.0 | 74.3 | 53 | 7.7 | 5.9 | 9.9 | 326 | 46.6 | 42.4 | 50.9 |
| **Smoking-related Health Diagnoses** |  |  |  |  |  |  |  |  |  |  |  |  |  |  |  |  |  |  |  |  |  |
| High blood pressure (R01_AX0111_01) | 1 = Marked | 117 | 18.4 | 15.6 | 21.7 | 149 | 24.6 | 20.5 | 29.3 | 496 | 81.6 | 78.3 | 84.4 | 29 | 5.0 | 3.4 | 7.3 | 351 | 56.5 | 52.5 | 60.4 |
| HIgh cholesterol (R01_AX0111_02) | 1 = Marked | 100 | 23.0 | 19.3 | 27.2 | 134 | 30.9 | 25.8 | 36.5 | 341 | 77.0 | 72.8 | 80.7 | 30 | 7.0 | 4.7 | 10.3 | 232 | 52.8 | 48.2 | 57.4 |
| Other heart condition (R01_AX0111_06) | 1 = Marked | 37 | 23.7 | 16.9 | 32.2 | 38 | 25.0 | 16.8 | 35.5 | 118 | 76.3 | 67.8 | 83.1 | ⱡ | | | | 86 | 54.5 | 46.7 | 62.2 |
| Chronic bronchitis (R01_AX0119_02) | 1 = Marked | 46 | 25.1 | 19.1 | 32.2 | 44 | 26.0 | 19.0 | 34.5 | 129 | 74.9 | 67.8 | 80.9 | ⱡ | | | | 79 | 46.1 | 38.4 | 53.9 |
| Asthma (R01_AX0119_04) | 1 = Marked | 86 | 24.2 | 19.1 | 30.1 | 63 | 18.6 | 14.2 | 24.0 | 268 | 75.8 | 69.9 | 80.9 | 18 | 5.1 | 3.1 | 8.3 | 186 | 51.8 | 45.9 | 57.6 |
| Cancer (R01_AX0144) | 1 = Yes | 33 | 26.1 | 19.0 | 34.7 | 30 | 22.6 | 15.9 | 31.0 | 102 | 73.9 | 65.3 | 81.0 | ⱡ | | | | 69 | 48.0 | 38.2 | 57.9 |
| **Nicotine dependence** | 0-33.3 | 51 | 15.9 | 12.1 | 20.6 | 41 | 12.2 | 9.2 | 16.1 | 295 | 84.1 | 79.4 | 87.9 | 14 | 4.8 | 2.8 | 8.0 | 217 | 58.5 | 52.4 | 64.4 |
| (R01R_A_DPNDSCL_NOMISS) | 33.4-66.7 | 220 | 21.9 | 19.0 | 25.2 | 168 | 17.8 | 15.2 | 20.7 | 803 | 78.1 | 74.8 | 81.0 | 54 | 5.6 | 4.0 | 7.7 | 590 | 56.9 | 52.7 | 61.1 |
|  | 66.8-100 | 295 | 27.4 | 24.6 | 30.4 | 233 | 23.0 | 19.8 | 26.5 | 778 | 72.6 | 69.6 | 75.4 | 94 | 8.3 | 6.8 | 10.1 | 524 | 48.7 | 45.1 | 52.3 |
| **Smoke-free home** (R01_AR1045) | 1 = Smoking is not allowed anywhere | 299 | 24.6 | 21.9 | 27.4 | 221 | 18.5 | 15.8 | 21.4 | 940 | 75.4 | 72.6 | 78.1 | 91 | 7.3 | 5.8 | 9.0 | 683 | 54.3 | 50.9 | 57.7 |
| **Perceived harm of cigarettes** | Not to somewhat harmful | 87 | 16.9 | 12.4 | 22.5 | 77 | 18.3 | 14.4 | 22.9 | 400 | 83.1 | 77.5 | 87.6 | 29 | 6.0 | 3.6 | 9.8 | 277 | 57.0 | 51.8 | 62.1 |
| (R01_AC9050) | Very/extremely harmful | 477 | 25.0 | 23.0 | 27.1 | 365 | 19.6 | 17.3 | 22.1 | 1476 | 75.0 | 72.9 | 77.0 | 133 | 6.8 | 5.8 | 8.1 | 1054 | 52.8 | 50.0 | 55.5 |
| **Relative perceived harm of e-cigarettes** | 1 = Less harmful | 375 | 31.1 | 28.1 | 34.3 | 203 | 17.2 | 14.7 | 20.1 | 859 | 68.9 | 65.7 | 71.9 | 117 | 9.4 | 7.7 | 11.4 | 576 | 45.1 | 41.6 | 48.6 |
| (R01_AE1099) | 2 = About the same | 159 | 18.0 | 15.2 | 21.2 | 169 | 20.8 | 17.8 | 24.1 | 743 | 82.0 | 78.8 | 84.8 | 37 | 4.6 | 3.2 | 6.6 | 543 | 59.5 | 55.5 | 63.4 |
|  | 3 = More harmful | 14 | 8.9 | 5.2 | 14.6 | 33 | 22.1 | 15.1 | 31.2 | 132 | 91.1 | 85.4 | 94.8 | ⱡ | | | | 107 | 71.8 | 62.9 | 79.2 |
| **Exposure to other smokers** | 0 | 26 | 15.1 | 10.0 | 22.1 | 49 | 28.0 | 21.3 | 36.0 | 158 | 84.9 | 77.9 | 90.0 | ⱡ | | | | 116 | 59.1 | 50.5 | 67.1 |
| (R01_AX0068) | 1-10 | 274 | 22.6 | 20.1 | 25.3 | 231 | 19.7 | 17.0 | 22.7 | 964 | 77.4 | 74.7 | 79.9 | 80 | 6.7 | 5.2 | 8.6 | 698 | 55.2 | 52.0 | 58.4 |
|  | >10 | 257 | 26.5 | 23.2 | 30.1 | 155 | 17.1 | 14.5 | 20.0 | 723 | 73.5 | 69.9 | 76.8 | 73 | 7.6 | 6.0 | 9.5 | 496 | 50.1 | 46.4 | 53.9 |
|  | Missing | ⱡ | | | | ⱡ | | | | ⱡ | | | | ⱡ | | | | 21 | 53.6 | 38.7 | 67.9 |
| **Age started smoking fairly regularly** | 18+ | 243 | 23.0 | 20.2 | 26.0 | 199 | 19.3 | 16.2 | 22.9 | 856 | 77.0 | 74.0 | 79.8 | 67 | 6.0 | 4.7 | 7.6 | 640 | 56.4 | 52.7 | 60.0 |
| (R01R_A_AGEREG_CIGS) | < 18 | 316 | 24.3 | 21.5 | 27.3 | 242 | 19.9 | 17.4 | 22.7 | 985 | 75.7 | 72.7 | 78.5 | 93 | 7.2 | 5.8 | 9.0 | 666 | 50.9 | 47.5 | 54.3 |
| **Cigarette consumption** | 1-9 CPD | 129 | 21.4 | 17.9 | 25.4 | 63 | 10.7 | 8.0 | 14.1 | 513 | 78.6 | 74.6 | 82.1 | 36 | 5.9 | 4.2 | 8.3 | 389 | 58.9 | 53.7 | 63.9 |
| (R01R_A_PERDAY_EDY_CIGS) | 10-19 CPD | 217 | 24.0 | 20.4 | 28.0 | 177 | 19.7 | 16.7 | 23.2 | 717 | 76.0 | 72.0 | 79.6 | 49 | 5.8 | 4.3 | 7.7 | 526 | 54.5 | 50.6 | 58.4 |
|  | 20+ CPD | 216 | 25.1 | 22.2 | 28.2 | 195 | 25.2 | 21.9 | 28.8 | 619 | 74.9 | 71.8 | 77.8 | 76 | 8.3 | 6.6 | 10.3 | 406 | 50.0 | 46.5 | 53.6 |
| **Pack-years of smoking** | <= 20 | 421 | 25.8 | 23.4 | 28.4 | 230 | 14.4 | 12.3 | 16.7 | 1262 | 74.2 | 71.6 | 76.6 | 121 | 7.2 | 6.0 | 8.8 | 926 | 53.8 | 50.5 | 57.0 |
| (R01R_A_PACKYEARS_CIGS) | 21-35 | 87 | 20.6 | 16.8 | 24.9 | 109 | 26.3 | 21.7 | 31.4 | 341 | 79.4 | 75.1 | 83.2 | 29 | 6.6 | 4.6 | 9.4 | 234 | 55.0 | 50.2 | 59.6 |
|  | > 35 | 54 | 18.3 | 14.2 | 23.1 | 96 | 33.2 | 27.6 | 39.2 | 246 | 81.7 | 76.9 | 85.8 | 11 | 4.3 | 2.6 | 7.3 | 161 | 53.8 | 47.7 | 59.7 |
| **Interest in quitting cigarettes** | 1-7 | 138 | 20.6 | 16.8 | 24.9 | 85 | 14.4 | 11.2 | 18.4 | 522 | 79.4 | 75.1 | 83.2 | 41 | 6.4 | 4.5 | 8.9 | 361 | 53.3 | 48.5 | 58.0 |
| (R01_AN0230) | 8-9 | 135 | 30.4 | 26.3 | 34.9 | 73 | 17.7 | 13.6 | 22.8 | 308 | 69.6 | 65.1 | 73.7 | 37 | 8.8 | 6.4 | 12.0 | 219 | 49.2 | 44.1 | 54.4 |
|  | 10 (extremely Interested) | 190 | 23.7 | 20.7 | 27.0 | 195 | 24.1 | 20.7 | 27.9 | 631 | 76.3 | 73.0 | 79.3 | 45 | 5.3 | 3.8 | 7.2 | 468 | 55.9 | 52.1 | 59.6 |
| **Self-efficacy about quitting** | No intent to quit in next 6 mos | 362 | 23.1 | 20.5 | 25.8 | 258 | 17.5 | 15.2 | 19.9 | 1225 | 76.9 | 74.2 | 79.5 | 116 | 7.3 | 6.0 | 8.8 | 853 | 52.9 | 49.7 | 56.1 |
| (R01_AN0245) | Not at all or a little likely | 35 | 23.7 | 17.8 | 30.9 | 33 | 22.3 | 15.1 | 31.5 | 125 | 76.3 | 69.1 | 82.2 | ⱡ | | | | 94 | 56.4 | 47.2 | 65.1 |
|  | Somewhat likely | 103 | 25.8 | 21.6 | 30.4 | 91 | 21.9 | 17.5 | 27.1 | 315 | 74.2 | 69.6 | 78.4 | 19 | 4.3 | 2.7 | 6.8 | 230 | 53.1 | 47.6 | 58.5 |
|  | Very likely | 66 | 22.6 | 17.1 | 29.4 | 59 | 23.7 | 18.0 | 30.4 | 209 | 77.4 | 70.6 | 82.9 | ⱡ | | | | 152 | 55.9 | 50.0 | 61.7 |
| **Length of LQA reported at W1** | < 30 d | 137 | 26.8 | 23.0 | 31.0 | 120 | 23.6 | 19.6 | 28.2 | 384 | 73.2 | 69.0 | 77.0 | 34 | 6.3 | 4.2 | 9.4 | 286 | 54.5 | 49.8 | 59.1 |
| (R01R_A_DURQUIT) | 30+ d | 50 | 27.7 | 20.5 | 36.3 | 42 | 25.8 | 19.8 | 32.8 | 127 | 72.3 | 63.7 | 79.5 | ⱡ | | | | 92 | 49.9 | 41.9 | 57.9 |
|  | No available data | 379 | 22.1 | 19.8 | 24.5 | 280 | 17.4 | 15.3 | 19.7 | 1366 | 77.9 | 75.5 | 80.2 | 117 | 6.8 | 5.7 | 8.1 | 953 | 53.6 | 50.7 | 56.6 |
| **Timing of LQA** | < 6 mo | 108 | 29.2 | 23.7 | 35.4 | 72 | 20.2 | 16.4 | 24.7 | 257 | 70.8 | 64.6 | 76.3 | 42 | 11.7 | 8.3 | 16.2 | 208 | 55.9 | 50.1 | 61.6 |
| (R02R_A_DAYSUNTILQA_CIGS) | 6+ mo | 213 | 26.6 | 23.2 | 30.2 | 159 | 21.5 | 17.9 | 25.5 | 593 | 73.4 | 69.8 | 76.8 | 75 | 9.2 | 7.2 | 11.7 | 468 | 58.3 | 53.8 | 62.6 |
|  | No available data | 245 | 19.8 | 17.5 | 22.3 | 211 | 17.7 | 15.1 | 20.5 | 1027 | 80.2 | 77.7 | 82.5 | 45 | 3.6 | 2.5 | 5.0 | 655 | 49.8 | 46.5 | 53.1 |
| *Abbrevations*: CPD, Cigarettes per day; FPL, Federal Poverty Level; LQA, last quit attempt; Wtd % Weighted Percent  † Estimate was suppressed because it has low statistical precision. It is based on a denominator sample size of less than 50, or the coefficient of variation of the estimate or its complement is larger than 30%. | | | | | | | | | | | | | | | | | | | | | |

# Supplement 3. Improvement in Covariate Balance with Propensity Score Matching: Comparability of Groups (Kernel Density Plots and Average Covariate Balance Grid across all Bootstrap runs)

Boxplots show, for each covariate in the propensity score model, the bootstrap distribution of the mean difference between exposed and non-exposed samples, before matching (left hand plot) and after matching (right hand plot), across all bootstrap samples (1500 bootstrap for comparisons 1 & 2, 2000 bootstrap samples for comparisons 3&4). For each bootstrap sample, the covariate is standardized using the entire sample prior to dividing into exposed and non-exposed subjects and taking the mean. Missing observations are imputed for each bootstrap sample as an initial step. The bootstrap samples are drawn with replacement and so sometimes our matched sample will be higher than the whole sample on the variable of interest.

eFigure 1. Comparison 1: E-cigarette on LQA versus no E-cigarette on LQA

| E-cigarette on LQA vs no E-cigarette on LQA: Randomly selected example from 1500 Bootstrap runs (PS of e-cigarette use) | |
| --- | --- |
| 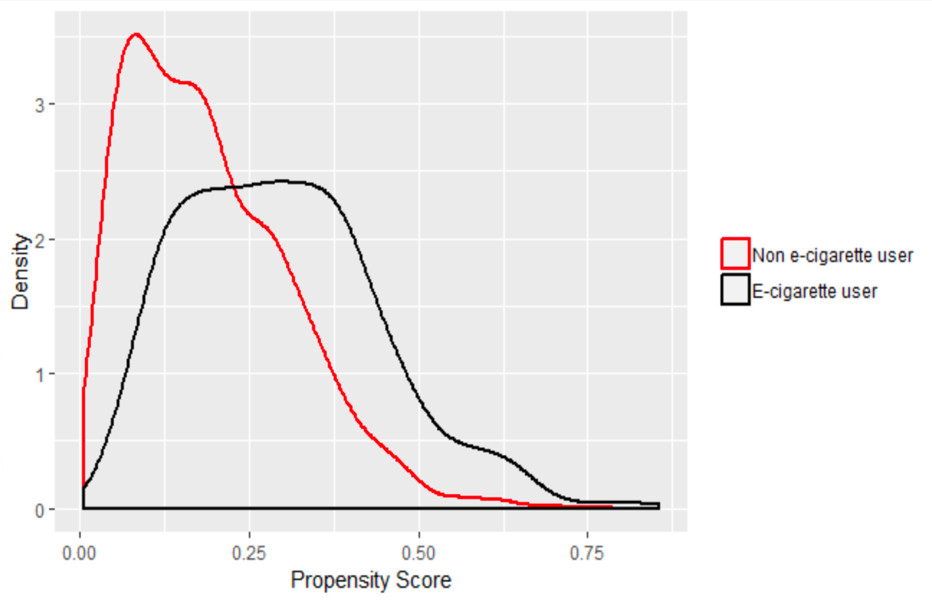 | 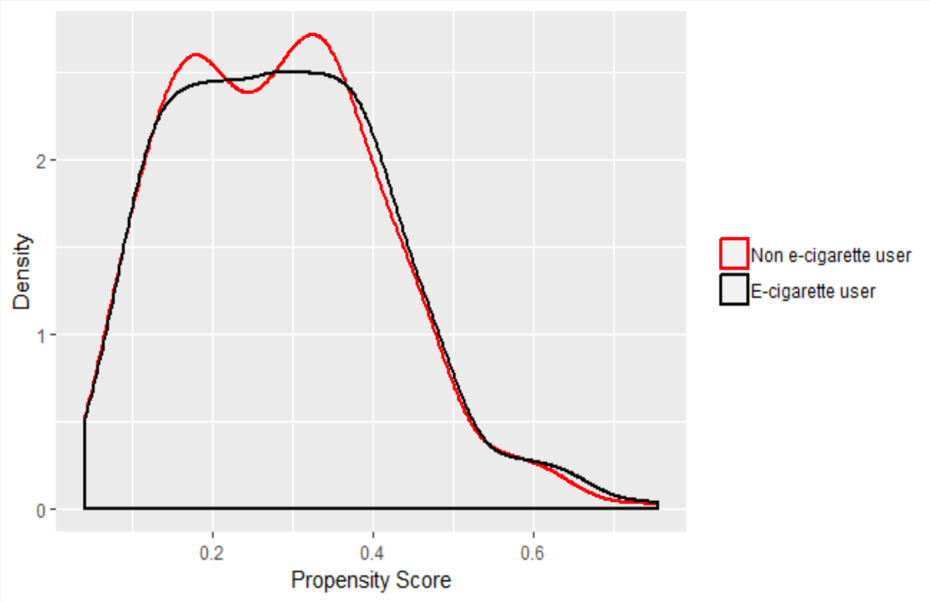 |
| Whole Sample Size (E-cigarettes: 562, non-E-cigarettes: 1844, numbers differ from Table 2 due missing values in smoke covariates | After Matching (E-cigarettes: 498, non-E-cigarettes: 498, matching is without replacement) |
| 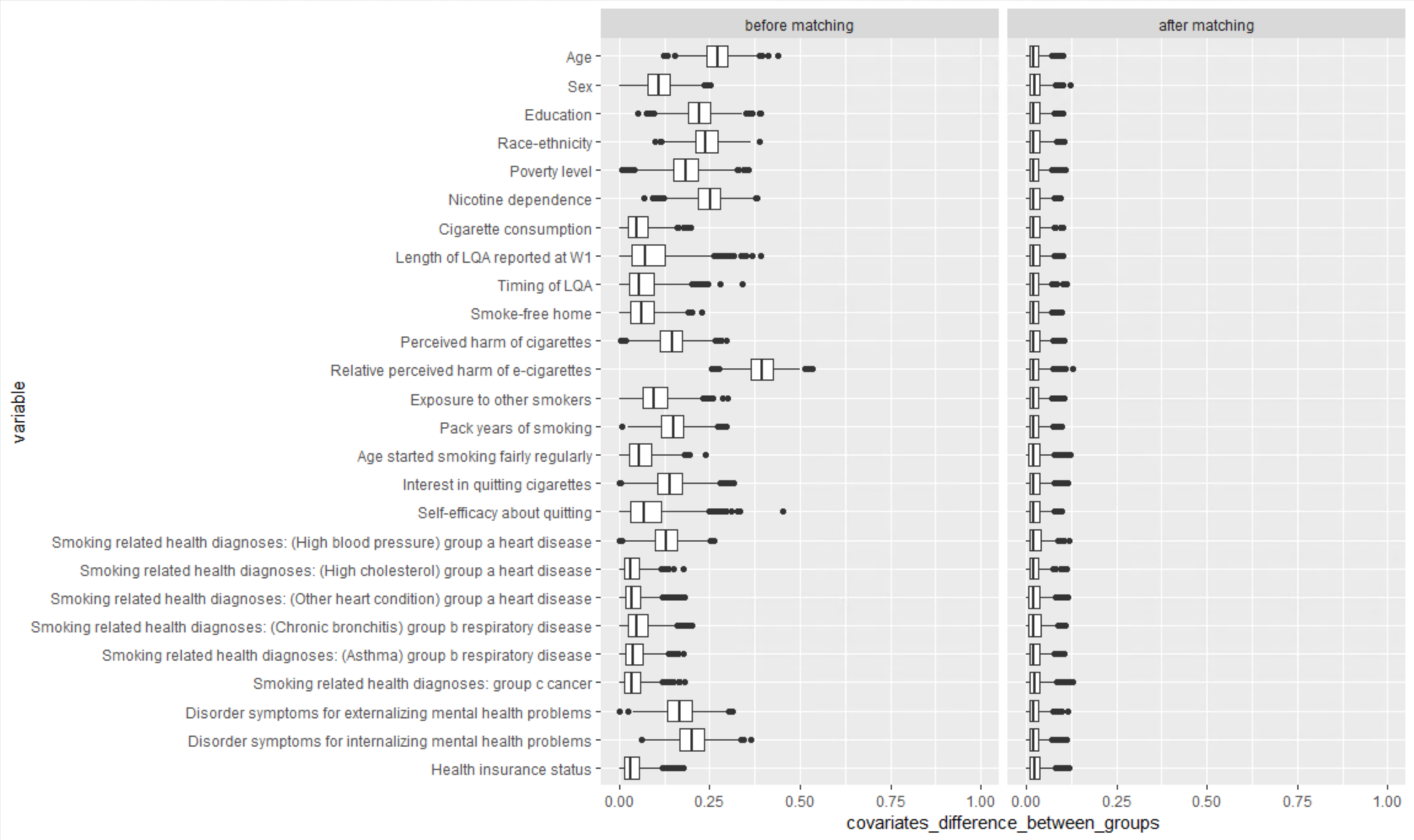  For a given covariate, we define “a marked improvement in covariate balance from matching” as a decrease of at least 0.1 units in the median difference of the standardized covariate between exposed and non-exposed subjects, comparing the bootstrap distribution before and after matching. These comparisons do not use the survey weights. For comparison 1, the following 12 covariates below achieved a marked improvement in covariate balance from the matching procedure (ordered by size of the difference in medians): Relative perceived of harm of e-cigarettes, age, nicotine dependence, race, education, disorder symptoms for internalizing mental health problems, poverty level, disorder symptoms for externalizing mental health problems, pack years of smoking, perceived harm of cigarettes, smoking related health diagnoses: (high blood pressure) group a heart disease, interest in quitting cigarettes. | |

eFigure 2. Comparison 2: E-cigarette on LQA versus no product on LQA

| E-cigarette on LQA vs no product on LQA: Randomly selected example from 1500 Bootstrap runs (PS of e-cigarette use) | |
| --- | --- |
| 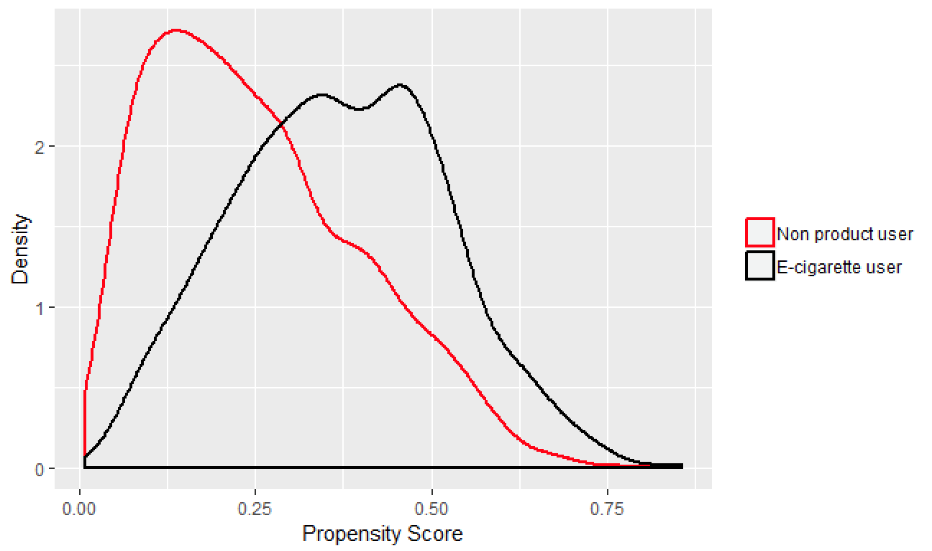 | 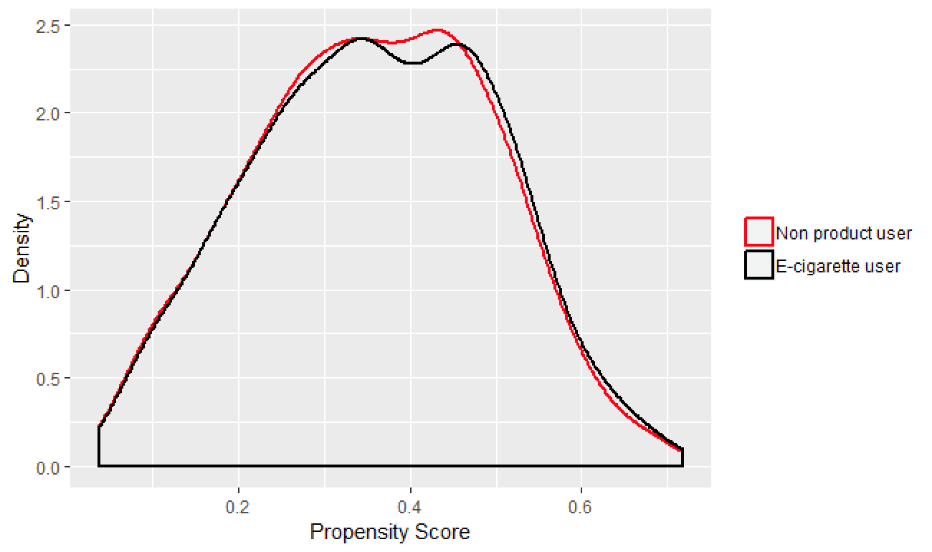 |
| Whole Sample Size (E-cigarettes:562, non-E-cigarettes:1387. Note these are slightly smaller than the totals in tables in study paper as there was some missing data in covariates) | After Matching (E-cigarettes: 535, non- E-cigarettes:535, matching is without replacement) |
| 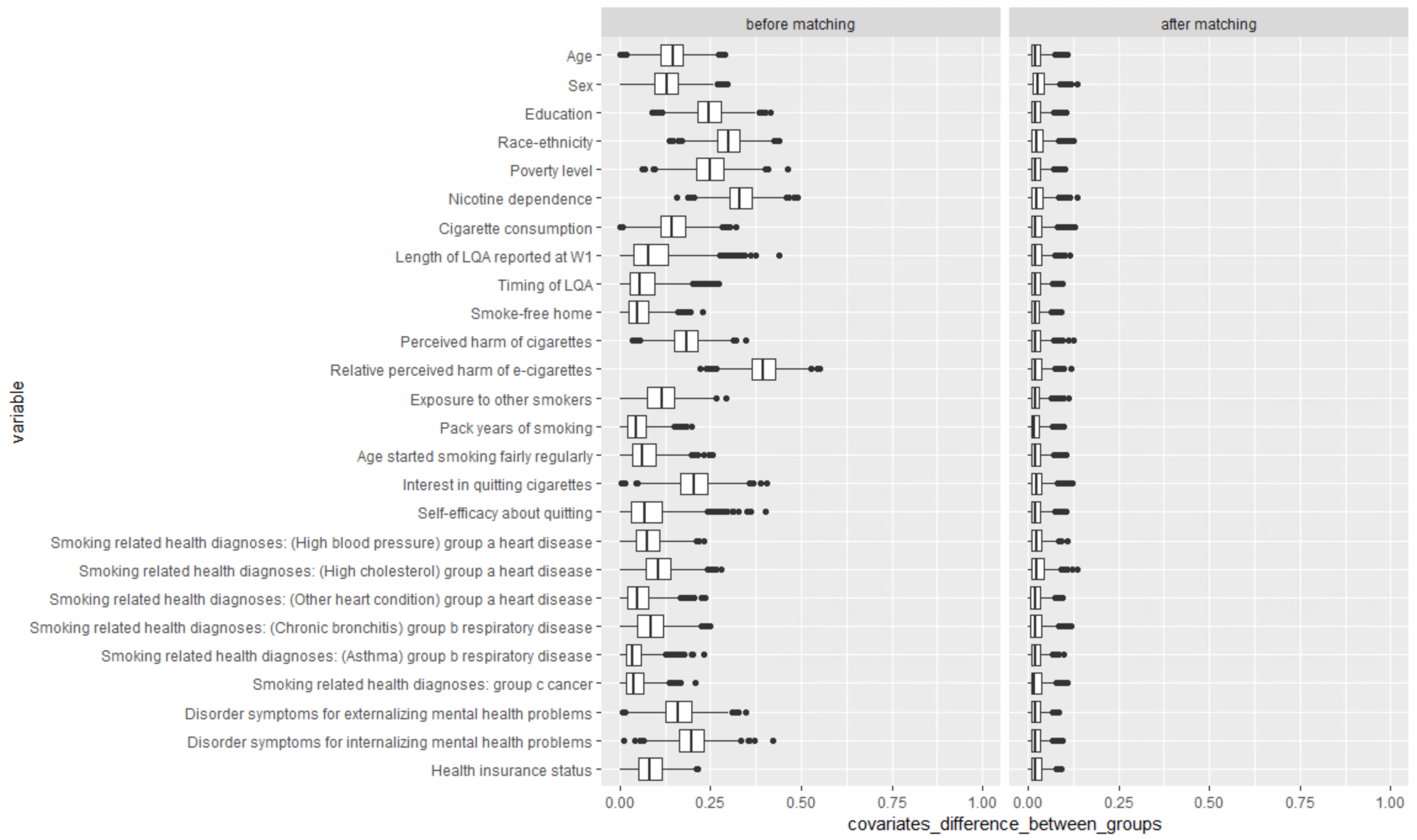  For comparison 2, the following 13 covariates achieved a marked improvement in covariate balance with the matching procedure across all bootstrap samples (ordered by size of the difference in medians): Relative perceived of harm of e-cigarettes, nicotine dependence, race, poverty level, education, interest in quitting cigarettes, disorder symptoms for internalizing mental health problems, perceived harm of cigarettes, disorder symptoms for externalizing mental health problems, cigarette consumption, age, gender, exposure to other smokers. | |

eFigure 3. Comparison 3: E-cigarette on LQA versus Any Pharmaceutical Aid for LQA

| E-cigarette on LQA vs Pharmaceutical on LQA: Randomly selected example from 2000 Bootstrap runs (propensity score of pharmaceutical aid use) | |
| --- | --- |
| 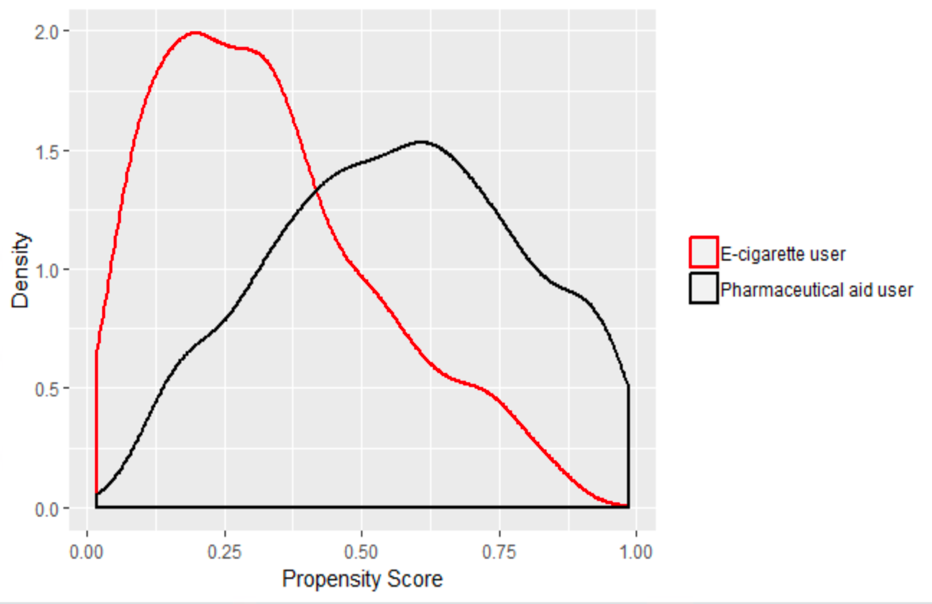 | 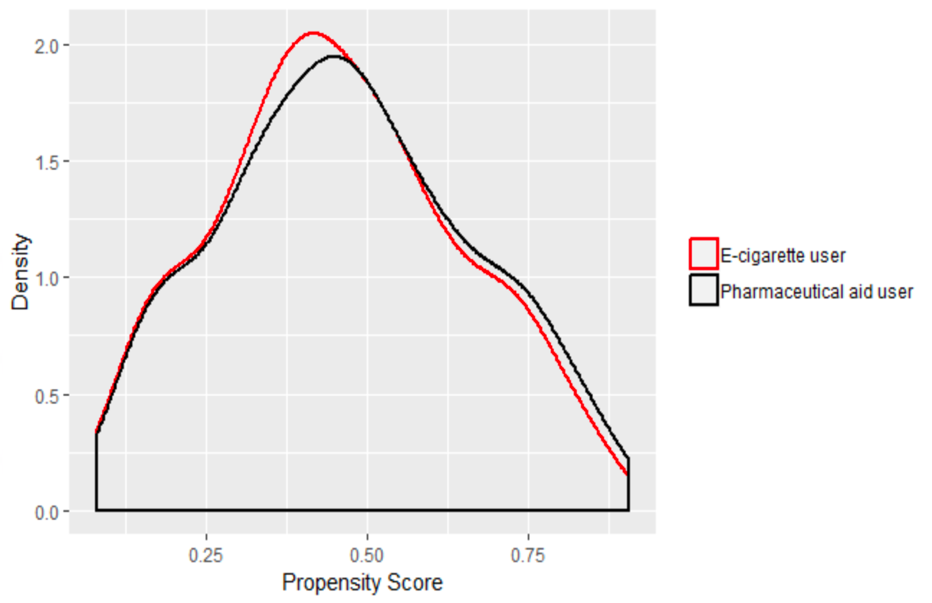 |
| Whole Sample Size (E-cigarettes:562,Pharma:437. Note these are slightly smaller than the totals in tables in study paper as there was some missing data in covariates.) | After Matching (E-cigarettes:291,Pharma:291, matching is without replacement) |
| 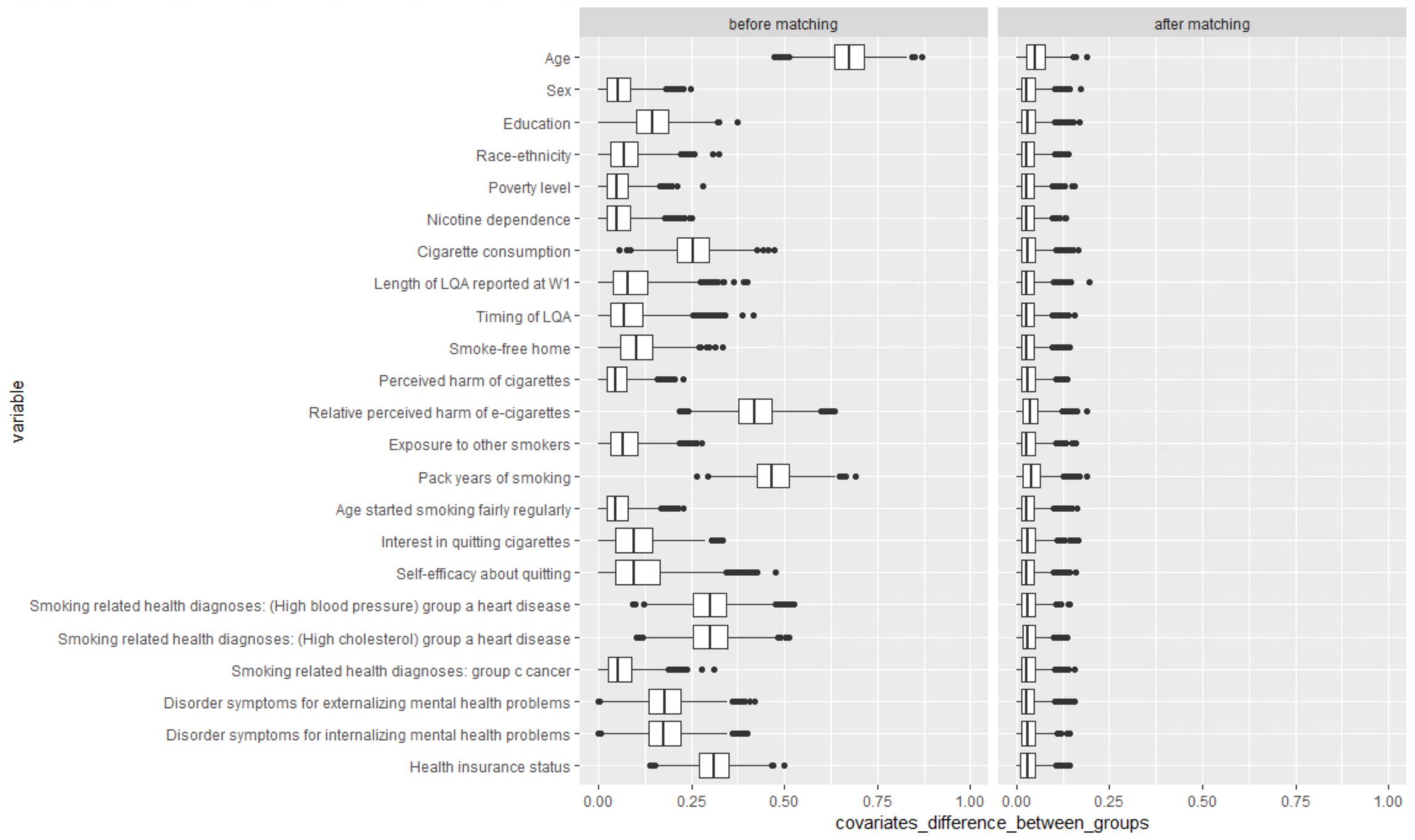 | |

For comparison 3, the following 10 covariates achieved a marked improvement in covariate balance from the matching procedure across all bootstrap samples (ordered by size of the difference in medians): age, pack years of smoking, relative perceived harm of e-cigarettes, smoking related health diagnoses: (high cholesterol) group a heart disease, smoking related health diagnoses: (high blood pressure) group a heart disease, health insurance status, cigarette consumption, disorder symptoms for internalizing mental health problems, disorder symptoms for externalizing mental health problems, education.

eFigure 4. Comparison 4: E-cigarette on LQA and daily e-cigarette at W2 versus no e-cigarette on LQA and W2

| E-cigarette on LQA and daily E-cigarette at W2 vs no E-cigarette on LQA or W2: Randomly selected example from 2000 Bootstrap runs (propensity score of daily e-cigarettes use) | |
| --- | --- |
| 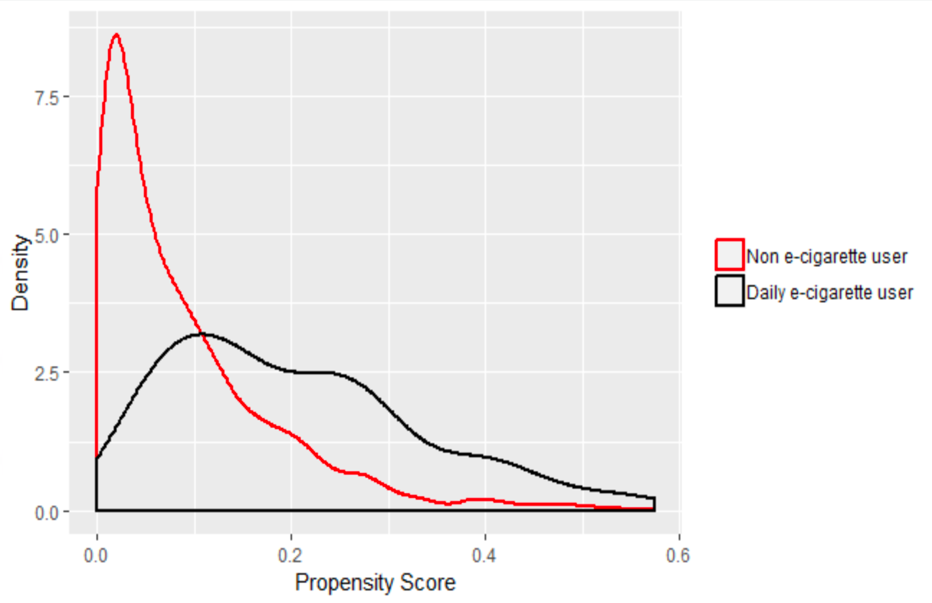 | 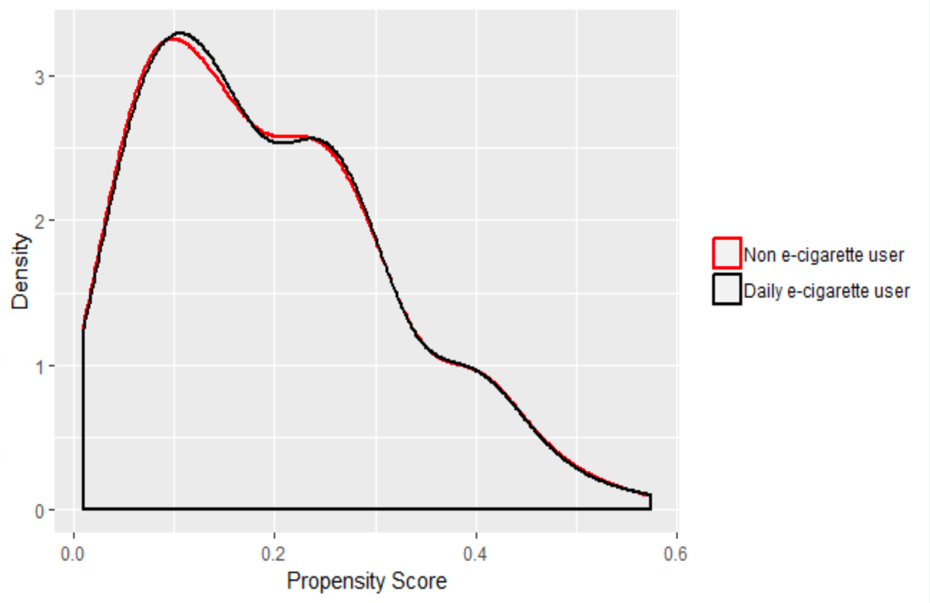 |
| Whole Sample Size (daily E-cigarettes:162, non-E-cigarettes:1573. Note these are slightly smaller than the totals in tables in study paper as there was some missing data in covariates.) | After Matching (daily E-cigarettes: 169, non- E-cigarettes:169. Note the matched daily e-cigarette sample of 169 is higher than the whole daily e-cigarette sample of 162. This occurs because the randomly selected bootstrap sample is drawn from the whole sample with replacement (i.e. daily e-cigarette users could be drawn multiple times from the whole sample).) |
| 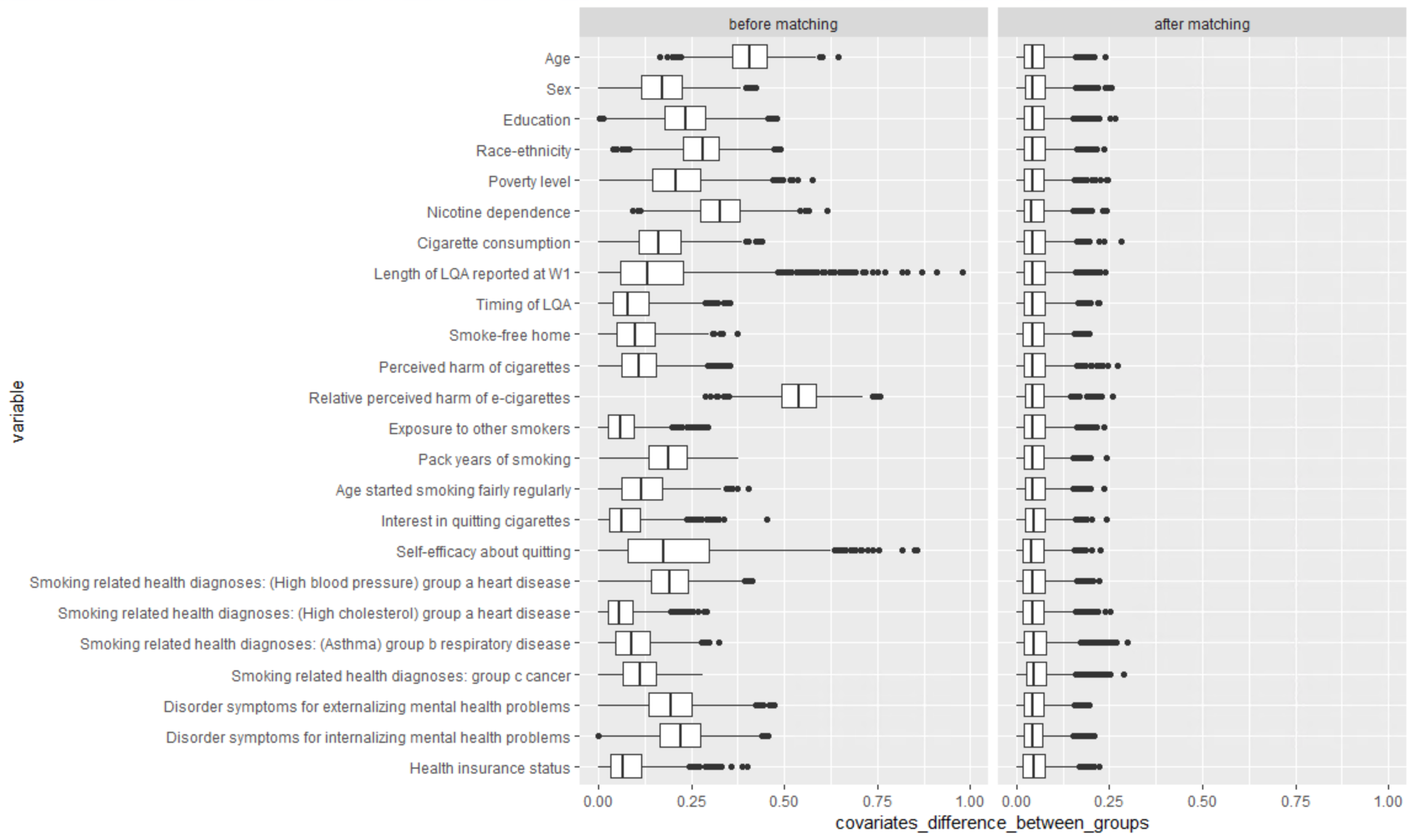  For comparison 4, the following 14 covariates achieved a marked improvement in covariate balance from the matching procedure across all bootstrap samples (ordered by size of the difference in medians): relative perceived harm of e-cigarettes, age, nicotine dependence, race, education, disorder symptoms for internalizing mental health problems, smoking related health diagnoses: (high blood pressure) group a heart disease, poverty level, disorder symptoms for externalizing mental health problems, pack years of smoking, self-efficacy about quitting, cigarette consumption, gender, age started smoking fairly regularly. | |

# Supplement 4. Sensitivity Analyses for PSM models

We present three components to these sensitivity analyses.

1. **The first (Main Sensitivity Analyses)** is the logistic regression analyses that parallel the PSM analyses.
2. **The second (Secondary Sensitivity Analyses 1)** stratifies the analyses on timing of the LQA, which is done for clarification purposes only
3. **The third (Secondary Sensitivity Analyses 2)** considers hypotheticals for daily e-cigarette use among last month quitters. Note that all sensitivity analyses use weighted data.

### a. Main Sensitivity Analyses

**Comparison 1. E-cigarette on LQA vs no e-cigarette on LQA:** Logistic regression of e-cigarette compared to no e-cigarette on the LQA, controlled for relevant propensity score covariates (age, gender, race, education level and nicotine dependence (ND)), corrected for multiple comparisons.

| eTable 2. E-cigarette on LQA vs no e-cigarette on LQA | | | | |
| --- | --- | --- | --- | --- |
|  | **Risk Difference** | **Risk Ratio** | **Odds Ratio** | **95% CI for OR** |
| 12+ months abstinence at W3 | 0.01 | 1.17 | 1.33 | (0.75, 2.35) |
| 30+ days abstinence at W3 | 0.02 | 1.18 | 1.25 | (0.80, 1.95) |

**Comparison 2. E-cigarette on LQA vs no product on LQA:** Logistic regression of e-cigarette use compared to no product, controlled for relevant propensity score covariates (age, gender, race, education level and nicotine dependence (ND)), corrected for multiple comparisons.

| eTable 3. E-cigarette on LQA vs no product on LQA | | | | |
| --- | --- | --- | --- | --- |
|  | **Risk Difference** | **Risk Ratio** | **Odds Ratio** | **95% CI for OR** |
| 12+ months abstinence at W3 | 0.01 | 1.14 | 1.29 | (0.71, 2.36) |
| 30+ days abstinence at W3 | 0.02 | 1.15 | 1.23 | (0.79, 1.91) |

**Comparison 3. E-cigarette on LQA vs Pharmaceutical on LQA:** Logistic regression of e-cigarette use compared to pharmaceutical aid, controlled for relevant propensity score covariates (age, gender, race, education level and nicotine dependence (ND)), corrected for multiple comparisons.

| eTable 4. E-cigarette on LQA vs no Pharmaceutical on LQA | | | | |
| --- | --- | --- | --- | --- |
|  | **Risk Difference** | **Risk Ratio** | **Odds Ratio** | **95% CI for OR** |
| 12+ months abstinence at W3 | 0.02 | 1.36 | 1.39 | (0.63, 3.08) |
| 30+ days abstinence at W3 | 0.04 | 1.28 | 1.26 | (0.64, 2.46) |

**Comparison 4. Daily e-cigarette use at W2 (and e-cigarette use on LQA) vs no e-cigarette on LQA or W2**: Logistic regression of daily e-cigarette at W2 and e-cigarette use on LQA compared to no e-cigarette use, controlled for relevant propensity score covariates (age, gender, race, education level, nicotine dependence (ND), abstinence 30+ days at W2, interaction between daily e-cigarette use and abstinence 30+ days at W2), corrected for multiple comparisons.

| eTable 5. Daily e-cigarette use at W2 (and e-cigarette use on LQA) vs no e-cigarette on LQA or W2 | | | | |
| --- | --- | --- | --- | --- |
| **4a. Among those abstinent 30+ days at W2** | **Risk Difference** | **Risk Ratio** | **Odds Ratio** | **95% CI for OR** |
| 12+ months abstinent at W3 | -0.07 | 0.85 | 0.90 | (0.38, 2.15) |
| 30+ days abstinent at W3 | -0.04 | 0.93 | 0.94 | (0.38, 2.32) |
| **4b. Among those not abstinent for 30+ days at W2** | | | | |
| 12+ months abstinent at W3 | 0.03 | 2.85 | 3.65 | (0.37, 36.35) |
| 30+ days abstinent at W3 | 0.09 | 2.12 | 2.43 | (1.08, 5.45) |

### Findings from the Main Set of Sensitivity Analyses

**Comparison 1. E-cigarette vs no e-cigarette on LQA:** There was no difference in either outcome (12+ month abstinence at W3 or 30+ day abstinence at W3) between those who used an e-cigarette on LQA and those who did not. The confidence limits on each odds ratio crossed 1.0.

**Comparison 2. E-cigarette vs no product on LQA:** There was no difference in either outcome between those who used an e-cigarette on LQA and those who did not use either an e-cigarette or a pharmaceutical aid (including NRT). The confidence limits on each odds ratio crossed 1.0.

**Comparison 3. E-cigarette vs Pharmacuetical aid (including NRT) on LQA:** There was no difference in either outcome between those who used an e-cigarette on LQA and those who used a pharmaceutical aid (including NRT). The confidence limits on each odds ratio crossed 1.0.

**Comparison 4. Daily e-cigarette use at W2 (and e-cigarette use on LQA) vs no e-cigarette on LQA or W2**

- **Strata a. Those abstinent 30+ days at W2**: There was no difference in either the 12+ month abstinence outcome or the 30+ day abstinence at W3 outcome between the daily e-cigarette group and those who did not use an e-cigarette on either the LQA or W2. The confidence limits for all odds ratios crossed 1.0.
- **Strata b. Those NOT abstinent for 30+ days at W2:** While there was no difference in the outcome of 12+ month abstinence at W3, there was a difference in the outcome of 30+ days abstinence at W3, with those who used an e-cigarette on the LQA more likely to be abstinent (OR=2.43, 95% C.I 1.08-5.45). This finding suggests that this group might be more likely to continue to progress toward quitting with another attempt that resulted in a 30+ day quit at W3.

### b. Secondary Sensitivity Analyses 1: Stratification on Timing of LQA

For these secondary sensitivity analyses, we conducted a series of logistic regressions which we stratified by the time of the last quit attempt (LQA) from W1, as some feel that this could be a potential confounder.

**Comparison 1. E-cigarette on LQA vs no e-cigarette on LQA**: Logistic regression of e-cigarette compared to no e-cigarette on the LQA, controlled for relevant propensity score covariates (age, gender, race, education level, nicotine dependence (ND), timing from W1 to LQA more than 9 months (with imputation - simple imputation with one imputed data set and assume a missing-at-random pattern - to handle missing data), interaction between e-cigarette use and timing from W1 to LQA more than 9 months), corrected for multiple comparisons.

| eTable 6. E-cigarette on LQA vs no e-cigarette on LQA | | | | |
| --- | --- | --- | --- | --- |
| **Among those** timing from W1 to LQA more than 9 months | **Risk Difference** | **Risk Ratio** | **Odds Ratio** | **95% CI for OR** |
| 12+ months abstinent at W3 | -0.01 | 0.86 | 0.94 | (0.25, 3.53) |
| 30+ days abstinent at W3 | -0.01 | 0.93 | 0.92 | (0.41, 2.03) |
| **Among those** timing from W1 to LQA less than 9 months |  |  |  |  |
| 12+ months abstinent at W3 | 0.03 | 1.36 | 1.60 | (0.85, 3.01) |
| 30+ days abstinent at W3 | 0.05 | 1.38 | 1.55 | (0.87, 2.76) |

**Comparison 2. E-cigarette on LQA vs no product on LQA:** Logistic regression of e-cigarettes compared to no product, controlled for relevant propensity score covariates (age, gender, race, education level and nicotine dependence (ND), timing from W1 to LQA more than 9 months (with imputation (simple imputation with one imputed data set and assume a missing-at-random pattern) to handle missing data), interaction between e-cigarette consumption and timing from W1 to LQA more than 9 months), corrected for multiple comparisons.

| eTable 7. E-cigarette on LQA vs no product on LQA | | | | |
| --- | --- | --- | --- | --- |
| **Among those** timing from W1 to LQA more than 9 months | **Risk Difference** | **Risk Ratio** | **Odds Ratio** | **95% CI for OR** |
| 12+ months abstinent at W3 | 0.002 | 1.03 | 1.18 | (0.37 3.78) |
| 30+ days abstinent at W3 | 0.0003 | 1.002 | 1.06 | (0.49, 2.26) |
| **Among those** timing from W1 to LQA less than 9 months |  |  |  |  |
| 12+ months abstinent at W3 | 0.02 | 1.23 | 1.38 | (0.67, 2.85) |
| 30+ days abstinent at W3 | 0.04 | 1.26 | 1.36 | (0.78, 2.37) |

**Comparison 3. E-cigarette on LQA vs Pharmaceutical on LQA:** Logistic regression of e-cigarette use compared to pharmaceutical aid, controlled for relevant propensity score covariates (age, gender, race, education level and nicotine dependence (ND), timing from W1 to LQA more than 9 months (with imputation (simple imputation with one imputed data set and assume a missing-at-random pattern) to handle missing data), interaction between e-cigarette consumption and timing from W1 to LQA more than 9 months), corrected for multiple comparisons.

| eTable 8. E-cigarette on LQA vs Pharmaceutical on LQA | | | | |
| --- | --- | --- | --- | --- |
| **Among those** timing from W1 to LQA more than 9 months | **Risk Difference** | **Risk Ratio** | **Odds Ratio** | **95% CI for OR** |
| 12+ months abstinent at W3 | 0.02 | 1.31 | 1.27 | (0.26 6.31) |
| 30+ days abstinent at W3 | 0.04 | 1.35 | 1.28 | (0.46, 3.61) |
| **Among those** timing from W1 to LQA less than 9 months |  |  |  |  |
| 12+ months abstinent at W3 | 0.03 | 1.39 | 1.41 | (0.49, 4.05) |
| 30+ days abstinent at W3 | 0.03 | 1,25 | 1.21 | (0.47, 3.12) |

**Comparison 4. Daily e-cigarette use at W2 (and** **e-cigarette use on LQA) vs no e-cigarette on LQA or W2**: Logistic regression of daily e-cigarette at W2 and e-cigarette use on LQA compared to no e-cigarette use, controlled for relevant propensity score covariates (age, gender, race, education level, nicotine dependence (ND)) and stratified by both timing from W1 to LQA (more than 9 months – i.e. close to W2) and abstinence 30+ days at W2, corrected for multiple comparisons.

| eTable 9. Daily e-cigarette use at W2 (and e-cigarette use on LQA) vs no e-cigarette on LQA or W2 | | | | | |
| --- | --- | --- | --- | --- | --- |
| **a. Among those abstinent 30+ days at W2** | | **Risk Difference** | **Risk Ratio** | **Odds Ratio** | **95% CI for OR** |
| Among those timing from W1 to LQA more than 9 months | 12+ months abstinent at W3 | -0.23 | 0.55 | 0.52 | (0.09,3.11) |
|  | 30+ days abstinent at W3 | -0.22 | 0.66 | 0.49 | (0.07,3.18) |
| Among those timing from W1 to LQA less than 9 months | 12+ months abstinent at W3 | 0.02 | 1.04 | 1.22 | (0.37,3.95) |
|  | 30+ days abstinent at W3 | 0.06 | 1.10 | 1.37 | (0.42,4.42) |
| **b. Among those not abstinent for 30+ days at W2** | |  |  |  |  |
| Among those timing from W1 to LQA more than 9 months | 12+ months abstinent at W3 | 0.02 | 2.51 | 2.87 | (0.04,211.54) |
|  | 30+ days abstinent at W3 | 0.04 | 1.44 | 1.54 | (0.33,7.27) |
| Among those timing from W1 to LQA less than 9 months | 12+ months abstinent at W3 | 0.03 | 3.12 | 4.33 | (0.21,88.02) |
|  | 30+ days abstinent at W3 | 0.13 | 2.85 | 3.44 | (1.17,10.12) |

### Findings from Secondary Sensitivity Analyses 1

**Comparison 1. E-cigarette vs no e-cigarette on LQA**: Regardless of whether the LQA was made in the first 9 months after W1 or the remaining time to W2, there was no difference in either outcome (12+ month abstinence at W3 or 30+ day abstinence at W3) between those who used an e-cigarette to quit and those who did not. The confidence limits on each odds ratio crossed 1.0.

**Comparison 2. E-cigarette vs no product on LQA**: Regardless of whether the LQA was made in the first 9 months after W1 or the remaining time to W2, there was no difference in either outcome between those who used an e-cigarette to quit and those who did not use either an e-cigarette or a pharmaceutical aid (including NRT). The confidence limits on each odds ratio crossed 1.0.

**Comparison 3. E-cigarette vs Pharmacuetical aid (including NRT) on LQA:** Regardless of whether the LQA was made in the first 9 months after W1 or the remaining time to W2, there was no difference in either outcome between those who used an e-cigarette to quit and those who used a pharmaceutical aid (including NRT). The confidence limits on each odds ratio crossed 1.0.

**Comparison 4. Daily e-cigarette use at W2 (and e-cigarette use on LQA) vs no e-cigarette on LQA or W2**

**Strata a. Among those quit 30+ days at W2**: Regardless of whether the LQA was made in the first 9 months after W1 or the remaining time to W2, there was no difference between in either outcome between those who were daily e-cigarette users at W2 and those who did not use an e-cigarette on either the LQA or W2. The confidence limits for all odds ratios crossed 1.0.

**Strata b. Among those not quit 30+ days at W2**: There was no difference by timing of the LQA on the outcomes of 12+ month cigarette abstinence at W3. However, timing of the LQA made a difference for the outcome 30+ days abstinence at W3. This difference was limited to those with a last quit attempt in the first 9 months of the W1-W2 period. Thus, all of this group were relapsers at W2. Among this subgroup, those who used an e-cigarette on the LQA were more likely to make another quit attempt and be quit for 30+ days at W3 (OR=3.44, 95% C.I. 1.17-10.12).

### c. Secondary Sensitivity Analyses 2: Hypotheticals among E-cigarette users with LQA <1 month prior to W2

Goal: To estimate the association of daily e-cigarette use for quitting with long-term cigarette abstinence. Unfortunately, the PATH Study has an estimate of daily e-cigarette use at Wave 2, but not for the last quit attempt. By limiting our consideration to those who quit in the month prior to Wave 2, W2 daily e-cigarette is quite proximal to the recalled quit attempt and so we can use maximal and minimal scenarios.

**Scenario 1**: Assume the PATH Study resembles the experience observed in the recently published randomized controlled trial of the efficacy of e-cigarette use for cessation (NEJM, 2019). In this trial, 53% of the e-cigarette group still used e-cigarettes daily at 4 weeks

Biases suggesting that 50% daily use at 4 wks may be higher than in population

- UK study advertised for participants and only randomized 43% of them
- Study used an adherence-enhancing intervention (4 sessions in 4 weeks)

Bias suggesting that 50% continued daily use may be too low for this PATH Study sample

- In the PATH Study, for those with a quit attempt in past month, the median time from the LQA is 9 days (95% C.I. 7-16 days) which is much less than 4 weeks

If we assume that these biases cancel out then the daily use of e-cigarettes at W2 will represents 50% of those who initially used e-cigarettes daily to help them quit. In this PATH Study sample, such a scenario means that all those who said that they used an e-cigarette to quit were daily users at the beginning of their quit attempt.

**Scenario 2:** We assume a new intervention for all those who started as daily users of e-cigarettes to help them quit which keeps all as daily users through W2. So daily use at W2 is the sum total of all those who ever used e-cigarettes daily for quitting.

### Unadjusted Population Estimates for Long-Term Cigarette Abstinence at W3

| eTable 10. Population estimates for who are cigarette abstinent for 12+ months at W3 | | |
| --- | --- | --- |
| **Product used on LQA** | **% Cigarette Abstinent for 12+m** | **95% C.L** |
| Scenario 1: all e-cig users were daily | **4.3%** | 1.0-16.1% |
| Scenario 2: W2 daily were only dailys | **7.0%** | 1.6- 26.6% |
| Control Group 1: No product on LQA | **8.2%** | 3.3- 19.0% |
| Control Group 2: Used pharma aid | **14.7%** | 6.6- 21.5% |

**Logistic Regression Sensitivity Analysis**

**Scenario 1: Used e-cigarette on LQA (n=56) vs no e-cigarette on LQA (n=168)**: Logistic regression of e-cigarette use compared to no e-cigarette use on the LQA, controlled for relevant propensity score covariates (age, gender, race, education level and nicotine dependence (ND)), corrected for multiple comparisons.

| eTable 11. E-cigarette on LQA vs no e-cigarette on LQA | | | | |
| --- | --- | --- | --- | --- |
|  | **Risk Difference** | **Risk Ratio** | **Odds Ratio** | **95% CI for OR** |
| **12+ months abstinence at W3** | -0.07 | 0.38 | **0.42** | (0.05, 3.31) |
| **30+ days abstinence at W3** | -0.17 | 0.43 | **0.43** | (0.19, 0.96) |

**Scenario 2: Daily W2 and e-cigs on LQA (n=28) versus no e-cigs on LQA (n=168)**: Controlled for relevant propensity score covariates (age, gender, race, education level and nicotine dependence (ND)), corrected for multiple comparisons.

| eTable 12. E-cigarette on LQA vs no e-cigarette on LQA | | | | |
| --- | --- | --- | --- | --- |
|  | **Risk Difference** | **Risk Ratio** | **Odds Ratio** | **95% CI for OR** |
| **12+ months abstinence at W3** | -0.04 | 0.59 | **0.70** | (0.08 5.82) |
| **30+ days abstinence at W3** | -0.07 | 0.74 | **1.01** | (0.36 2.83) |

### Findings from Secondary Sensitivity Analyses 2

Under each of these scenarios, there was no evidence to suggest that the population estimates for long-term quitting (12+ month) at W3 might be better for e-cigarette users. The logistic regression found no difference in either outcome (12+ month abstinence at W3 or 30+ day abstinence at W3) between those who used an e-cigarette to quit and those who did not. The confidence limits on each odds ratio crossed 1.0.

# Supplement 5. Timing of the Start of the Last Quit Attempt prior to Wave 2 by Product Used

Methods: Respondents who were quit at time of survey were asked how long it had been since they had last smoked. For those who were still quit at W2, we used this variable to calculate the time from the start of LQA to W2 survey. Respondents who had relapsed were asked the date the LQA ended; to get the timing of the start of the LQA we used this variable plus the reported length of quit. Some respondents who had returned to smoking did not report an end date of their quit attempt, these were dropped from this analysis; the n reported is those included in the analyses. Weighted median length of quit and 95% confidence limits were calculated using PROC SURVEYMEANS in SAS.

| eTable 13. Time from LQA to W2 by product, overall and by 3 time intervals | | | | | | |
| --- | --- | --- | --- | --- | --- | --- |
| Timing of LQA from W2 | Product Group | N | % of total* (unwtd) | Timing of LQA from W2 (Median) | 95 % CL (Lower) | 95% CL (Upper) |
| < 1 mo | Did not use e-cigs to quit | 168 | 75.0 | **14** | 10 | 17 |
|  | Used e-cigs to quit | 56 | 25.0 | **9** | 5 | 13 |
|  | Used e-cig to quit, daily user | 28 | 12.5 | **12** | 7 | 17 |
|  | Used pharma to quit | 47 | 21.0 | **12** | 7 | 16 |
|  | No product to quit | 118 | 52.7 | **14** | 10 | 19 |
| 2-6 mo | Did not use e-cigs to quit | 429 | 73.1 | **91** | 83 | 100 |
|  | Used e-cigs to quit | 158 | 26.9 | **93** | 76 | 111 |
|  | Used e-cig to quit, daily user | 53 | 9.0 | **91** | 68 | 113 |
|  | Used pharma to quit | 116 | 19.8 | **89** | 64 | 115 |
|  | No product to quit | 308 | 52.5 | **91** | 81 | 102 |
| 6+ mo | Did not use e-cigs to quit | 390 | 72.9 | **312** | 292 | 332 |
|  | Used e-cigs to quit | 145 | 27.1 | **294** | 272 | 317 |
|  | Used e-cig to quit, daily user | 47 | 8.8 | **271** | 226 | 315 |
|  | Used pharma to quit | 102 | 19.1 | **304** | 272 | 336 |
|  | No product to quit | 277 | 51.8 | **309** | 279 | 339 |
| Overall | Did not use e-cigs to quit | 987 | 73.3 | **135** | 125 | 146 |
|  | Used e-cigs to quit | 359 | 26.7 | **150** | 132 | 169 |
|  | Used e-cig to quit, daily user | 128 | 9.5 | **120** | 87 | 154 |
|  | Used pharma to quit | 265 | 19.7 | **131** | 106 | 155 |
|  | No product to quit | 703 | 52.2 | **132** | 115 | 148 |
|  | All Groups | 1346 | 100.0 | **138** | 129 | 148 |

*Note: These product groups are not mutually exclusive. The first two groups for each

time period are mutually exclusive and so the combined % of total for these two is 100%
